# Modeling of Static Stress Identification Using Electromechanical Impedance of Embedded Piezoelectric Plate

**DOI:** 10.3390/s24217096

**Published:** 2024-11-04

**Authors:** Xianfeng Wang, Hui Liu, Guoxiong Liu, Dan Xu

**Affiliations:** 1 School of Civil Engineering and Architecture, Wuhan University of Technology, Wuhan 430070, China; xfwang91@whut.edu.cn; 2 Sanya Science and Education Innovation Park, Wuhan University of Technology, Sanya 572025, China; 3 China Railway 11th Bureau Group Corporation Limited, Wuhan 430064, China; zt11jlgx@163.com (G.L.); xudan.11g@crcc.cn (D.X.)

**Keywords:** initial stress, static stress, electromechanical impedance, piezoelectricity, piezoelectric transducer

## Abstract

Working stress is an important indicator reflecting the health status of structures. Passive-monitoring technology using the piezoelectric effect can effectively monitor the dynamic stress of structures. However, under static loads, the charge generated by the piezoelectric devices can only be preserved when the external circuit impedance is infinitely large, which means passive-monitoring techniques are unable to monitor static and quasi-static stress caused by slow-changing actions. In current studies, experimental observations have shown that the impedance characteristics of piezoelectric devices are affected by external static loads, yet the underlying mechanisms remain inadequately explained. This is because the impedance characteristics of piezoelectric devices are actually dynamic characteristics under alternating voltage. Most existing impedance analysis models are based on linear elastic dynamics. Within this framework, the impact of static stress on dynamic characteristics, including impedance characteristics, cannot be addressed. Accounting for static stress in impedance modeling is a challenging problem. In this study, the static stress applied on an embedded piezoelectric plate is abstracted as the initial stress of the piezoelectric plate. Based on nonlinear elastic dynamic governing equations, using the displacement method, an impedance analysis model of an embedded piezoelectric plate considering initial stress is established and verified through a fundamental experiment and a finite element analysis. Based on this, the explicit analytical relation between initial stress and impedance characterizations is provided, the mechanism of the effect of initial stress on the impedance characterizations is revealed, and procedures to identify static stress using impedance characterizations is proposed. Moreover, the sensitivities of the impedance characterizations in response to the initial stress are thoroughly discussed. This study mainly provides a theoretical basis for monitoring static stress using the electromechanical impedance of an embedded piezoelectric plate. And the results of the present study can help with the performance prediction and design optimization of piezoelectric-based static stress sensors.

## 1. Introduction

As civil engineering structures develop toward a larger size and higher complexity, the safety of structures, especially the safety of infrastructure, has received worldwide attention. Structural health monitoring technology is an effective means to ensure the safety and stable service of engineering structures [1,2,3].

Working stress is an important indicator reflecting the health status of structures. However, the current stress monitoring methods have various limitations [4]: resistance strain gauges are not suitable for long-term monitoring, differential resistive sensors and vibrating wire sensors cannot monitor dynamic stress, fiber Bragg grating sensors and their supporting facilities are expensive, etc.

Piezoelectric materials, especially piezoelectric ceramics, are widely used in electromechanical conversion systems as energy transducers in various engineering fields [5,6,7,8,9,10]. In the field of structural health monitoring, there are three main types of techniques: the wave analysis technique [11,12,13,14,15], impedance analysis technique [16,17,18,19,20,21,22,23], and passive-monitoring technique [24,25,26,27,28,29]. Currently, passive-monitoring techniques utilizing the piezoelectric effect have enabled the dynamic stress monitoring of structures [26,27,28,29]. However, under static loads, the charge generated by piezoelectric devices can only be preserved when the external circuit impedance is infinitely large. This means passive-monitoring techniques are unable to monitor static and quasi-static stresses caused by slow-changing actions, such as prestressing in a prestressed structure, the temperature effect of large-volume concrete, earth pressure, etc.

While passive-monitoring techniques utilizing the piezoelectric effect cannot monitor static stresses, studies have shown that the impedance characteristics of piezoelectric devices are affected by applied static loads [20,21,22,23]. Monitoring static stress in structures through the impedance characteristics of embedded piezoelectric devices is a novel approach. In the research conducted by Zhang et al., piezoelectric plates were embedded in concrete specimens, and the impedance analysis method was used to monitor the load-bearing capacity degradation of the concrete specimens during axial compressive failure. The experimental loading process revealed that even in the elastic stage, the impedance characteristics of the piezoelectric plates were affected by the applied static loads [20]. Based on statistical analysis of experimental results, Pan and Guan proposed a nondestructive method to monitor the stress–strain relationship of concrete using embedded piezoelectric sensors. And the experimental results indicated that as the load increased, the measured conductance of the embedded piezoelectric sensors decreased in the applicable frequency [21]. Using a two-dimensional embedded PZT-structure interaction model, Ai et al. proposed a real-time identification approach for flexure-critical stress and damage in reinforced concrete beams. Both their analytical and experimental results showed that the conductance peak and resonant frequency of the embedded PZT transducer shifted under tension and compression stresses [22]. In real-scale structures, the impedance of implanted piezoelectric transducers is also found to be affected by applied static load. In the investigation conducted by Karayannis et al., implanted PZT transducers were employed to identify the damage level of a real-scale reinforced concrete beam–column joint. In this real-scale experiment, the PZT transducers were successfully used in damage diagnosis, and the impedance of the implanted PZT transducers was apparently affected by the applied static load [23].

The aforementioned studies have indeed demonstrated the feasibility of utilizing the impedance analysis method to monitor static stress from an experimental perspective. However, the underlying mechanisms remain inadequately explained. This is because the impedance characteristics of piezoelectric devices are actually dynamic characteristics under alternating voltage. Most existing impedance analysis models are based on linear elastic dynamics. Within this framework, the impact of static stress on dynamic characteristics, including impedance characteristics, cannot be addressed. These inadequate impedance analysis models, which do not account for static stress or incorrectly consider static stress as the stress boundary amplitude, make it impossible to accurately predict the performance and optimize the design of piezoelectric-based static stress sensors.

Accounting for static stress in impedance modeling is a challenging problem. Fortunately, in the field of solid mechanics, scholars have utilized the continuum mechanics approach to investigate the influence of initial stress on the elastic wave propagation characteristics of piezoelectric materials. They have established nonlinear elastic dynamic governing equations (constitutive equations, geometric equations, and equilibrium equations) of piezoelectric materials under the influence of initial stress, considering geometric nonlinearity [30,31,32]. It is worth noting that the “initial stress” here is not a boundary condition, but rather, an “initial condition” acting on the equilibrium equation prior to the application of dynamic loads. From the above review, it is evident that in seeking an impedance analysis model that considers the influence of applied static stress, the “applied static stress” may be abstracted as the “initial stress” of piezoelectric materials. Based on the nonlinear elastic dynamic governing equations of piezoelectric materials, an impedance analysis model that accounts for the influence of initial stress could be established.

In the present study, electromechanical impedance characterizations of an embedded piezoelectric plate are utilized to identify the internal static stress of structures. The internal static stress of structures applied on the embedded piezoelectric plate is considered to be the initial stress of the piezoelectric plate, and the impedance characterizations of the piezoelectric plate that change with the initial stress are employed to identify the initial stress, thereby enabling the identification of static stress. Specifically, based on the nonlinear elastic dynamic governing equations of piezoelectric materials, using the displacement method, an impedance analysis model of an embedded piezoelectric plate considering the effect of initial stress is established and verified through a fundamental experiment and a finite element analysis. Using this impedance analysis model, the explicit analytical relation between initial stress and impedance characterizations is determined, the mechanism of the effect of initial stress on the impedance characterizations is revealed, and a procedure to identify static stress is proposed. Moreover, the sensitivities of the impedance characterizations in response to the initial stress are thoroughly discussed. This study mainly provides a theoretical basis for monitoring static stress using the electromechanical impedance of an embedded piezoelectric plate. And the results of the present study can help with the performance prediction and design optimization of piezoelectric-based static stress sensors.

The rest of this paper is organized as follows. In Section 2, adopting a nonlinear dynamic equilibrium equation accounting for initial stress, an impedance analysis model of an embedded piezoelectric plate considering initial stress is established. In Section 3, a fundamental experiment and a finite element analysis are conducted to validate the proposed theoretical model. In Section 4, numerical studies are conducted to firstly reveal the effect of static stress on the impedance characterizations. Secondly, to evaluate an individual’s potential in static stress identification, the sensitivities of different characterizations to static stress are identified and compared to each other. Subsequently, to guide the design of piezoelectric-based static stress sensors, the impact of geometrical and mechanical factors on the sensitivities of impedance characterizations to static stress is thoroughly discussed. Section 5 concludes this study.

## 2. Impedance Analysis Model of an Embedded Piezoelectric Plate Considering Initial Stress

**Figure 1:**
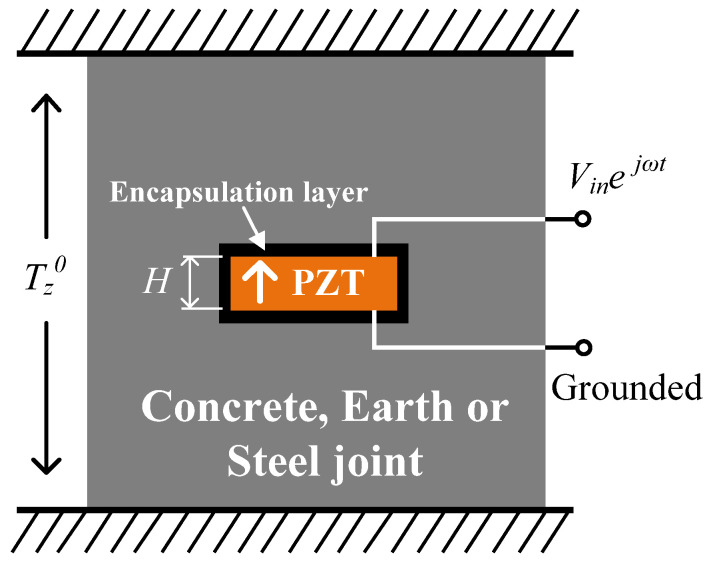
A piezoelectric plate embedded in an engineering structure.

**Figure 2:**
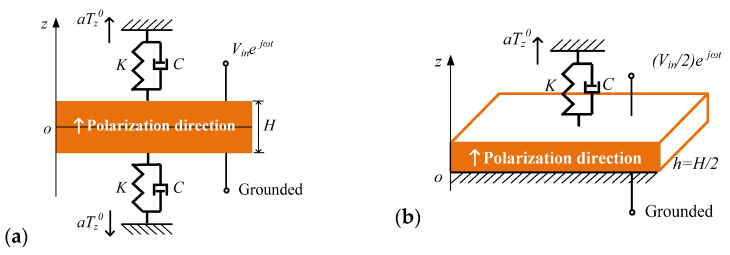
Simplified model of embedded piezoelectric plate: (**a**) full-scale model, and (**b**) semi-thickness model.

As shown in Figure 1, a piezoelectric plate covered with continuous electrodes on the upper and lower surfaces and encapsulated by insulating material is embedded in an engineering structure. The surrounding environment of the piezoelectric plate is reinforced concrete, foundation soil or a steel joint. Due to the external static load applied on the engineering structure, the embedded piezoelectric plate is subjected to static stress $T_{z}^{0}$ through the surrounding media. Note that a positive value of $T_{z}^{0}$ indicates tensile stress, while a negative value of $T_{z}^{0}$ indicates compressive stress. The upper surface of the embedded piezoelectric plate is subjected to a harmonic external voltage $v\left(t\right)=V_{in}e^{j\omega t}$, and the lower surface is grounded. Simplified models of the embedded piezoelectric plate are illustrated in Figure 2. In the simplified full-scale model presented in Figure 2a, the cross-sectional area of the plate is denoted as a, and the thickness of the plate is represented by *H*. As shown in Figure 2a, the piezoelectric plate is sandwiched by a pair of viscoelastic constraints with stiffness of *K* and a viscous damping coefficient of *C*. The piezoelectric plate is subjected to a static load $aT_{z}^{0}$ through the viscoelastic constraint ($T_{z}^{0}$ is the internal static stress of the structure, where a positive number indicates tensile stress and a negative number indicates compressive stress). Herein, the viscoelastic constraint is a simplification of the surrounding environment, and the static stress $T_{z}^{0}$ is considered to be the initial stress of the piezoelectric plate. As for the electrical loading, the lower surface of the piezoelectric plate is grounded, while the upper surface is subjected to a harmonic external voltage $v\left(t\right)=V_{in}e^{j\omega t}$. From the perspective of the displacement field, the simplified full-scale model presented in Figure 2a is a symmetrical structure subjected to symmetrical loads, which means it can be further simplified to a semi-thickness model, as presented in Figure 2b. The semi-thickness model is actually the upper half of the full-scale model with adjusted electrical boundary conditions. In the semi-thickness model, a semi-thickness piezoelectric plate with a thickness of h=H/2 is bonded on a rigid surface. The upper surface of the plate is subjected to a static tensile load $aT_{z}^{0}$ through the viscoelastic constraint, and the static stress $T_{z}^{0}$ is considered to be the initial stress of the piezoelectric plate. As for the adjusted electrical boundary conditions, the lower surface of the piezoelectric plate is grounded, while the upper surface is subjected to a harmonic external voltage $v\left(t\right)=\left(V_{in}/2\right)e^{j\omega t}$. Using the semi-thickness model, the mechanical and electrical components of the embedded piezoelectric plate can be obtained. It should be noted that from the perspective of circuit connection, the full-scale model consists of two semi-thickness model connected in series. This indicates that the full-scale model and the semi-thickness model have the same input current but different input voltages. In the following impedance characterization of the embedded piezoelectric plate, the input voltage of the full-scale model and the input current of the semi-thickness model are adopted.

### 2.1. Basic Equations

From the perspective of piezoelectricity, the constitutive equations of a piezoelectric plate polarized along the *z* axis can be written as [33]

(1) $$T_{z}=c_{33}^{E}S_{z}-e_{33}E_{z}$$

(2) $$D_{z}=e_{33}S_{z}+\varepsilon_{33}^{S}E_{z}$$

where $S_{z}$ and $T_{z}$ are the strain and stress components of the piezoelectric layer along the *z* direction, respectively. $E_{z}$ and $D_{z}$ are the electric field strength and the electric displacement along the *z* direction, respectively. $c_{33}^{E}$, *e*_33_ and $\varepsilon_{33}^{S}$ are the elastic stiffness, the piezoelectric constant and the dielectric constant, respectively. The superscripts *E* and *S* indicate a parameter at a constant electric field and constant strain, respectively. In the following derivation, the superscripts are dropped for simplicity. The geometric relationship equations of the piezoelectric layer can be expressed as

(3) $$S_{z}=\frac{\partial w}{\partial z}$$

(4) $$E_{z}=-\frac{\partial \phi }{\partial z}$$

where *w* is the displacement of the piezoelectric layer along the *z* direction, and *ϕ* is the electric potential. It is assumed that there exists only one constant initial stress component $T_{z}^{0}$ in the piezoelectric layer, such that the dynamic and electric displacement equilibrium equations of the piezoelectric layer can be written as

(5) $$\rho \frac{\partial^{2}w}{\partial t^{2}}=\frac{\partial T_{z}}{\partial z}+T_{z}^{0}\frac{\partial^{2}w}{\partial z^{2}}$$

(6) $$\frac{\partial D_{z}}{\partial z}=0$$

where *ρ* is the density of the piezoelectric layer. Herein, different from the conventional linear elastic model, a nonlinear dynamic equilibrium equation accounting for the initial stress term $T_{z}^{0}$, i.e., Equation (5), is employed [30,31,32]. If the input voltage *v*(*t*) is harmonic, it is of the form

(7) $$v\left(t\right)=V_{in}e^{j\omega t}$$

where $V_{in}$ is the input voltage amplitude, $j=\sqrt{-1}$ is the imaginary unit, ω=2πf is the circular frequency, and *f* is the input voltage frequency (driving frequency). The steady-state expressions of the stress, electric displacement, electric potential and displacement of the piezoelectric layer can be expressed as

(8) $$\left(T_{z},D_{z},\phi ,w\right)=\left[T_{z}\left(z\right),D_{z}\left(z\right),\phi \left(z\right),w\left(z\right)\right]e^{j\omega t}$$

Substituting Equations (3) and (4) into Equation (1) yields

(9) $$T_{z}\left(z\right)=\frac{c_{33}\partial w\left(z\right)}{\partial z}+\frac{e_{33}\partial \phi \left(z\right)}{\partial z}$$

Differentiate both sides of Equation (9) with respect to *z*:

(10) $$\frac{\partial T_{z}\left(z\right)}{\partial z}=\frac{c_{33}\partial^{2}w\left(z\right)}{\partial z^{2}}+\frac{e_{33}\partial^{2}\phi \left(z\right)}{\partial z^{2}}$$

Substituting Equations (3) and (4) into Equation (2) yields

(11) $$D_{z}\left(z\right)=\frac{e_{33}\partial w\left(z\right)}{\partial z}-\frac{\varepsilon_{33}\partial \phi \left(z\right)}{\partial z}$$

Substituting Equation (11) into Equation (6) yields

(12) $$\frac{\varepsilon_{33}\partial^{2}\phi \left(z\right)}{\partial z^{2}}=\frac{e_{33}\partial^{2}w\left(z\right)}{\partial z^{2}}$$

If we combine Equations (10) and (12), we have

(13) $$\frac{\partial T_{z}\left(z\right)}{\partial z}=\frac{\left({e_{33}}^{2}+c_{33}\varepsilon_{33}\right)}{\varepsilon_{33}}\frac{\partial^{2}w\left(z\right)}{\partial z^{2}}$$

Substituting Equation (13) into Equation (5) yields

(14) $$-\rho \omega^{2}w\left(z\right)=\frac{\left({e_{33}}^{2}+c_{33}\varepsilon_{33}\right)}{\varepsilon_{33}}\frac{\partial^{2}w\left(z\right)}{\partial z^{2}}+T_{z}^{0}\frac{\partial^{2}w\left(z\right)}{\partial z^{2}}$$

Equation (14) can be simplified as

(15) $$\frac{\partial^{2}w\left(z\right)}{\partial z^{2}}+\alpha^{2}w\left(z\right)=0$$

where $\alpha^{2}=\rho \omega^{2}/\left(\eta +T_{z}^{0}\right)$ and $\eta =\left({e_{33}}^{2}+c_{33}\varepsilon_{33}\right)/\varepsilon_{33}$. If we solve Equation (15), we have

(16) $$w\left(z\right)=A_{1}\sin\left(\alpha z\right)+A_{2}\cos\left(\alpha z\right)$$

where $A_{1}$ and $A_{2}$ are unknown constants. If we combine Equation (16) with Equation (12), we have

(17) $$\phi \left(z\right)=\beta \left[A_{1}\sin\left(\alpha z\right)+A_{2}\cos\left(\alpha z\right)+A_{3}z+A_{4}\right]$$

where $\beta =e_{33}/\varepsilon_{33}$, and $A_{3}$ and $A_{4}$ are integration constants. Using Equation (9), the stress component of the piezoelectric layer can be obtained as follows:

(18) $$T_{z}\left(z\right)=\alpha \eta \left[A_{1}\cos\left(\alpha z\right)-A_{2}\sin\left(\alpha z\right)\right]+\delta A_{3}$$

where $\eta =\left({e_{33}}^{2}+c_{33}\varepsilon_{33}\right)/\varepsilon_{33}$ and $\delta ={e_{33}}^{2}/\varepsilon_{33}$. Using Equation (11), the electric displacement of the piezoelectric layer can be obtained as follows:

(19) $$D_{z}\left(z\right)=-e_{33}A_{3}$$

Until now, the expressions of the stress, the electric displacement, the electric potential and the displacement of the piezoelectric layer have been obtained, and the unknown constants are $A_{1}$, $A_{2}$, $A_{3}$ and $A_{4}$. Note that these basic equations are valid for both the full-scale model and the semi-thickness model. The theoretical solutions of impedance characterization for the embedded piezoelectric plate can be obtained using the input voltage of the full-scale model and the input current of the semi-thickness model.

### 2.2. Theoretical Solutions of the Semi-Thickness Model

The electrical boundary conditions and the mechanical boundary conditions of the semi-thickness model, as shown in Figure 2b, are summarized as follows, respectively.

The electrical boundary conditions of the semi-thickness piezoelectric plate:

(20) $$\left\{\begin{array}{l} {\left.\phi \left(z\right)\right|}_{z=h}=\left(V_{in}/2\right)\\ {\left.\phi \left(z\right)\right|}_{z=0}=0 \end{array}\right.$$

The mechanical boundary conditions of the semi-thickness piezoelectric plate:

(21) $$\left\{\begin{array}{l} {\left.w\left(z\right)\right|}_{z=0}=0\\ {\left.aT\left(z\right)\right|}_{z=h}=-{Kw\left(z\right)|}_{z=h}-{j\omega Cw\left(z\right)|}_{z=h} \end{array}\right.$$

The above boundary conditions generate the following four independent linear algebraic equations:

(22) $$\left\{\begin{array}{l} \beta \sin\left(\alpha h\right)A_{1}+\beta \cos\left(\alpha h\right)A_{2}+\beta hA_{3}+\beta A_{4}=\left(V_{in}/2\right)\\ \beta A_{2}+\beta A_{4}=0\\ A_{2}=0\\ a\alpha \eta \left[A_{1}\cos\left(\alpha h\right)-A_{2}\sin\left(\alpha h\right)\right]+a\delta A_{3}\\ =-\left(K+j\omega C\right)\left[A_{1}\sin\left(\alpha h\right)+A_{2}\cos\left(\alpha h\right)\right] \end{array}\right.$$

And the four unknown constants $A_{1}$, $A_{2}$, $A_{3}$ and $A_{4}$ can be solved as follows:

(23) $$\left\{\begin{array}{l} A_{1}=\frac{\left(V_{in}/2\right)\delta a}{\beta \delta a\sin\left(\alpha h\right)-\beta h\left(K+j\omega C\right)\sin\left(\alpha h\right)-\alpha \beta \eta ha\cos\left(\alpha h\right)}\\ A_{2}=0\\ A_{3}=\frac{-\left[\alpha \eta a\cos\left(\alpha h\right)+\left(K+j\omega C\right)\sin\left(\alpha h\right)\right]A_{1}}{a\delta }\\ A_{4}=0 \end{array}\right.$$

Further, the input electric current of the semi-thickness model can be defined as

(24) $$i\left(t\right)=\frac{dq\left(t\right)}{dt}$$

where $q\left(t\right)$ is the input charge of the semi-thickness model given by

(25) $$q\left(t\right)=\int_{a}D_{z}\left(h\right)nda$$

where $D_{z}\left(h\right)$ and **n** are, respectively, the electric displacement and the inward unit normal vector of the ungrounded side surface. Here, we take n=−1. Substituting Equations (19) and (25) into Equation (24), the input electric current can be expressed as

(26) $$i\left(t\right)=I_{in}e^{j\omega t}=\omega ae_{33}A_{3}je^{j\omega t}$$

where $I_{in}=\omega ae_{33}A_{3}j$ is the amplitude phasor of the input electric current, and $\left|I_{in}\right|$ is the amplitude of the input electric current.

### 2.3. Theoretical Solutions of Impedance Characterizations for Embedded Piezoelectric Plate

Considering the perspective of circuit connection, the full-scale model consists of two semi-thickness models connected in series, which means the full-scale model has the same input current as the semi-thickness model. Using the input voltage $V_{in}$ of the full-scale model and the input current $I_{in}$ of the semi-thickness model, the input impedance *Z* and admittance *Y* of the embedded piezoelectric plate can be determined, respectively, as

(27) $$\left\{\begin{array}{l} Z=\frac{V_{in}}{I_{in}}=\frac{V_{in}}{\omega ae_{33}A_{3}j}\\ =\frac{2\beta \delta a\sin\left(\alpha h\right)-2\beta h\left(K+j\omega C\right)\sin\left(\alpha h\right)-2\alpha \beta \eta ha\cos\left(\alpha h\right)}{\omega ae_{33}\left[\alpha \eta a\cos\left(\alpha h\right)+\left(K+j\omega C\right)\sin\left(\alpha h\right)\right]}j\\ Y=\frac{1}{Z} \end{array}\right.$$

One should notice that the input impedance of the embedded piezoelectric plate illustrated by the full-scale model is exactly twice that of the semi-thickness model.

## 3. Model Validation

In Section 2, the explicit solution of the electromechanical impedance for an embedded piezoelectric plate considering the initial stress is obtained. In this section, the impedance analysis model presented in Section 2 is verified by comparing its simplification via a fundamental experiment and a finite element analysis.

### 3.1. Experimental Validation

**Figure 3:**
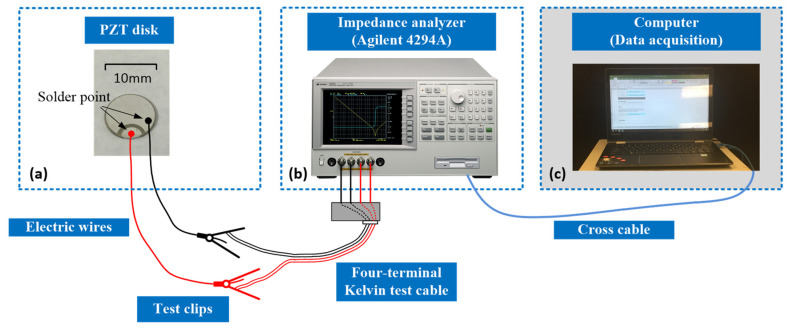
Experimental setup: (**a**) sample PZT disk, (**b**) impedance analyzer, and (**c**) data acquisition computer.

By taking $T_{z}^{0}=0$, K=0 and C=0, the present impedance analysis model is simplified to that of a free-boundary PZT disk and compared with the experimental impedance frequency response of three sample PZT disks. The sample disks and testing platform presented in Figure 3 were employed in one of our previous works [8].

An illustration of the employed PZT disks is presented in Figure 3a. The effective cross-sectional area, thickness, density and dielectric constant of these three sample PZT disks are very close to each other, and we take average values, respectively, of $a=6.17\times 10^{-5}\;m^{2}$, H=0.5 mm, $\rho =7600\;kg/m^{3}$ and $\varepsilon_{33}^{S}=1.02\times 10^{-8}\;F/m$. The sample PZT disks have asymmetrical electrodes, as shown in Figure 3a, and the shuttle-shaped part is connected to the round electrode on the backside. The effective cross-sectional area is considered to be the area of the smaller electrode. The dielectric constant is obtained using the capacitance of the sample PZT disk times H then divided by a.

The resonant frequency $f_{r}$, the anti-resonant frequency $f_{a}$, the elastic constant $c_{33}^{E}$ and the piezoelectric constant $e_{33}$ of these three sample PZT disks are presented in Table 1. It should be noted that $f_{r}$ and $f_{a}$ are identified from the experimentally obtained impedance frequency response, i.e., the driving frequency corresponding to the minimum impedance is identified as $f_{r}$, and the driving frequency corresponding to the maximum impedance is identified as $f_{a}$. Once $f_{r}$ and $f_{a}$ are identified, $c_{33}^{E}$ and $e_{33}$ can be resolved. The procedures to resolve $c_{33}^{E}$ and $e_{33}$ from $f_{r}$ and $f_{a}$ are provided in Appendix A.

**Figure 4:**
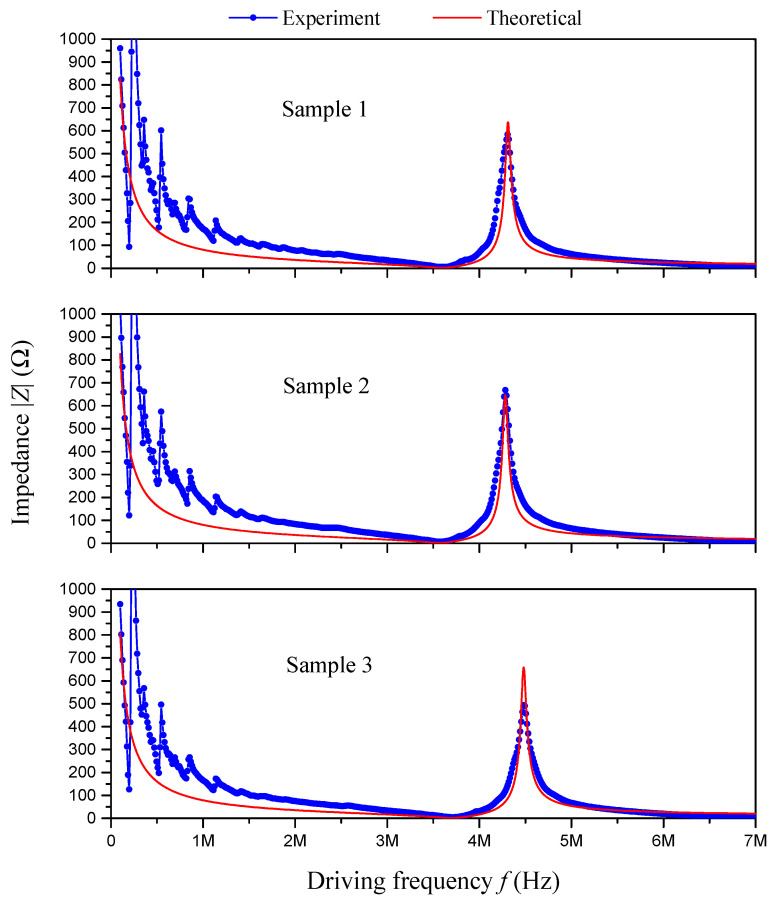
Impedance frequency response of sample PZT disks: experimental and theoretical results.

The impedance frequency responses of these three sample PZT disks are tested using an impedance analyzer, as presented in Figure 3b, the data are gathered using a computer, as presented in Figure 3c, and the results are presented in Figure 4.

In the simplification of the present proposed impedance analysis model, to consider the internal damping effect of the material, the elastic constant $c_{33}^{E}$ is replaced with $c_{33}^{E}\left(1+Qj\right)$, where *Q* is the internal complex damping factor and *j* is the imaginary unit. Theoretically, the larger the complex damping factor *Q*, the smaller the resonant displacement. The damping factor *Q* also affects the valley and peak values of the absolute impedance at the resonant and anti-resonant frequencies, respectively. That is to say, the larger the complex damping factor *Q*, the larger the valley value but the smaller the peak value of the absolute impedance. In the simplified impedance analysis model, we take Q=0.02.

The theoretical impedance frequency responses of the sample PZT disks obtained from the simplification of the present model are presented and compared with the experimental results in Figure 4. As we can see in Figure 4, for the thickness stretch mode, the resonant and anti-resonant frequencies obtained using the present model match well with the experimental results. The mismatch in the peak value of the absolute impedance is due to the estimation of the complex damping factor *Q*. Actually, the specific damping factor for each sample PZT disk can be identified using the present model if needed.

### 3.2. Finite Element Method Validation

The proposed impedance analysis model is preliminary validated through a fundamental experiment, and further verification considering the impact of initial stress is conducted using the finite element method (FEM) in the following section.

Herein, in both the theoretical and the finite element analysis, a numerical sample piezoelectric plate made from standard PZT-5H with a cross-sectional area of $a=100\;{mm}^{2}$ and a thickness of H=2 mm is employed. And the one-dimensional material parameters of standard PZT-5H are listed in Table 2.

**Figure 5:**
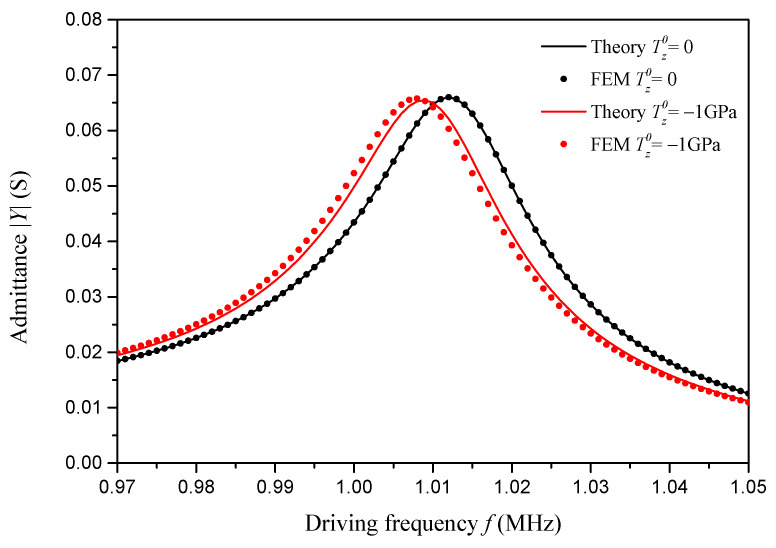
Admittance frequency response considering initial stress: FEM and theoretical results.

Based on the present impedance analysis model, using Equation (27), considering the internal complex damping factor and the external viscoelastic constraint, respectively, to be Q=0.02, K=0 and C=0, the theoretical admittance frequency responses of the sample piezoelectric plate with different initial stresses are determined and are presented in Figure 5 and compared with the finite element results. In this finite element method validation, two initial stresses are considered, i.e., $T_{z}^{0}=0$ and $T_{z}^{0}=-1\;GPa$.

The finite element results for validation are obtained using COMSOL Multiphysics 5.6. In the COMSOL project, the Solid Mechanics Module and the Electrostatic Module are employed, the plane strain assumption is adopted, a two-dimensional piezoelectric actuator model is created, and a frequency-domain perturbation study is conducted. To simulate one-dimensional performance using a two-dimensional model, the one-dimensional material parameters of PZT-5H listed in Table 2 are manually entered in the COMSOL project. As for the damping mechanism and initial stress, an isotropic elastic loss factor of 0.02 and a prestress term are adopted in the COMSOL project. The admittance frequency responses for no prestress and a 1 GPa compressive prestress obtained from the COMSOL project are presented in Figure 5 for verification. The COMSOL project is provided in the Supplementary Materials.

As we can see in Figure 5, when there is no initial stress, the theoretical admittance frequency response almost perfectly matches the finite element result. When a 1 GPa compressive initial stress is applied on the sample piezoelectric plate, both the theoretical and FEM curves shift leftward, resulting in a slight decrease in admittance peaks and an obvious decrease in resonant frequency. From Figure 5, we can conclude that the theoretically predicted impact of initial stress is consistent with the finite element result.

## 4. Numerical Results and Discussion

In this section, numerical studies are conducted to firstly reveal the effect of initial stress on the impedance characterizations of an embedded piezoelectric plate and secondly, to evaluate an individual’s potential in static stress identification. The sensitivities of different characterizations to the compressive initial stress are identified and compared to each other. Subsequently, procedures to identify static stress using impedance characterizations of an embedded piezoelectric plate are proposed. Moreover, to guide the design of piezoelectric-based static stress sensors, the effect of the geometric dimensions, the external constraint and the damping mechanism on the sensitivities of both the conductance peak and the resistance peak to the initial stress is thoroughly discussed.

The following numerical results are obtained using the explicit theoretical solutions presented in Equation (27). Specifically, all the frequency response curves are directly obtained from Equation (27), achieved using MATLAB R2017b with 1000 Hz driving frequency spacing. And the peak or valley values as well as corresponding resonant or anti-resonant frequencies are identified from the frequency response data. As for the sensitivities of impedance characterizations to initial stress, different initial stresses yield varying characterizations. We plot the scatters of the varying characterizations with respect to varying initial stresses, and the slope of the linear fitting line of these discrete scatters is identified as the sensitivity. In the following numerical study, the one-dimensional material parameters of standard PZT-5H are employed and listed in Table 2.

### 4.1. Effect of Initial Stress on Impedance Characterizations

An analysis of the frequency response of the impedance characterizations considering the effect of the initial stress is conducted in this section. Six impedance characterizations are taken into account, i.e., the absolute value of impedance $\left|Z\right|$, absolute value of admittance $\left|Y\right|$, resistance-$real\left(Z\right)$, reactance-$imag\left(Z\right)$, conductance-$real\left(Y\right)$ and susceptance-$imag\left(Y\right)$. In this part, a numerical sample piezoelectric plate made from standard PZT-5H with a cross-sectional area of $a=100\;{mm}^{2}$ and a thickness of H=2 mm is employed. As mentioned in Section 3, to consider the internal damping effect of the material, the elastic constant $c_{33}^{E}$ is replaced with $c_{33}^{E}\left(1+Qj\right)$, where *Q* is the complex damping factor and *j* is the imaginary unit. In this part, the internal damping factor of the material is considered to be Q=0.02, and the external viscoelastic constraint is considered to be K=0 and C=0.

To clearly show the effect of the initial stress on the impedance characterizations, extremely large compressive initial stresses are considered in the following numerical study, i.e., 0, −10 GPa, −20 GPa, −30 GPa, −40 GPa and −50 GPa. These considered initial stresses exceed the ultimate load-bearing capacity of PZT-5H. For much smaller initial stress in practice, the effect on impedance characterizations should have the same pattern, as illustrated in the following section.

**Figure 6:**
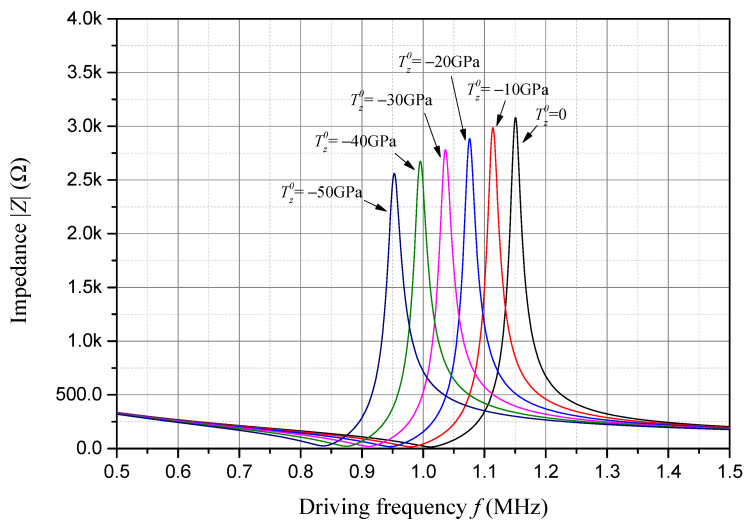
Impedance frequency response considering compressive initial stress.

The impedance frequency response of the sample piezoelectric plate considering six different compressive initial stresses is presented in Figure 6. The frequency corresponding to the peak value of the impedance is approximately the anti-resonant frequency of the sample piezoelectric plate. And the frequency corresponding to the valley value of the impedance is approximately the resonant frequency of the sample piezoelectric plate. As one can see in Figure 6, with an increase in the compressive initial stress, both the impedance peak and the corresponding anti-resonant frequency decrease linearly.

**Figure 7:**
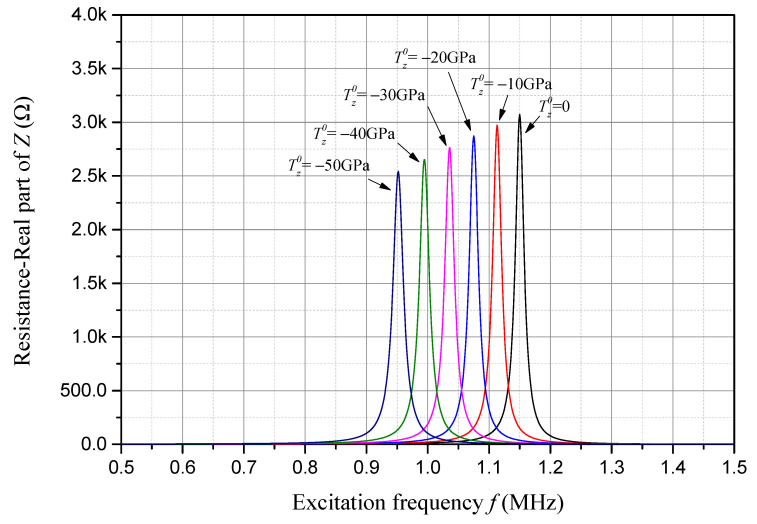
Resistance frequency response considering compressive initial stress.

Resistance is the real part of impedance, and its frequency response considering different compressive initial stresses is presented in Figure 7. The frequency corresponding to the peak value of the resistance is also approximately the anti-resonant frequency of the sample piezoelectric plate. As one can see in Figure 7, with an increase in the compressive initial stress, both the resistance peak and the corresponding anti-resonant frequency decrease linearly.

**Figure 8:**
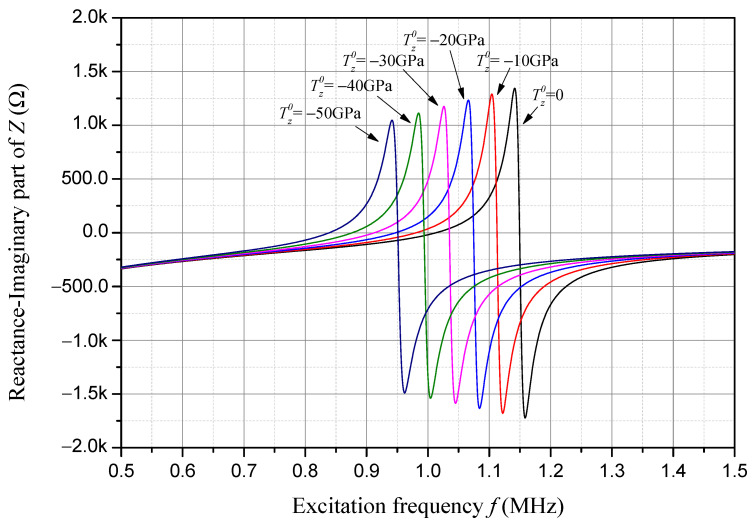
Reactance frequency response considering compressive initial stress.

Reactance is the imaginary part of impedance, and its frequency response considering different compressive initial stresses is presented in Figure 8. The frequencies where the reactance firstly and secondly approaches zero are, respectively, the exact resonant and anti-resonant frequencies of the sample piezoelectric plate. As one can see in Figure 8, with an increase in the compressive initial stress, the resonant frequency, the anti-resonant frequency, and the absolute peak and valley values of the reactance decrease linearly.

**Figure 9:**
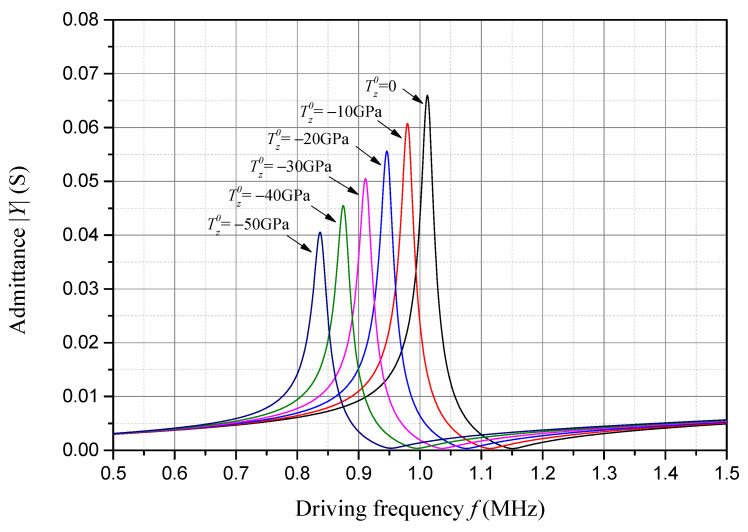
Admittance frequency response considering compressive initial stress.

For the same extremely large compressive initial stress, i.e., 0, −10 GPa, −20 GPa, −30 GPa, −40 GPa and −50 GPa, the admittance frequency response of the sample piezoelectric plate is presented in Figure 9. The frequency corresponding to the peak value of the admittance is approximately the resonant frequency of the sample piezoelectric plate. And the frequency corresponding to the valley value of the admittance is approximately the anti-resonant frequency of the sample piezoelectric plate. As one can see in Figure 9, with an increase in the compressive initial stress, both the admittance peak and the corresponding resonant frequency decrease linearly.

**Figure 10:**
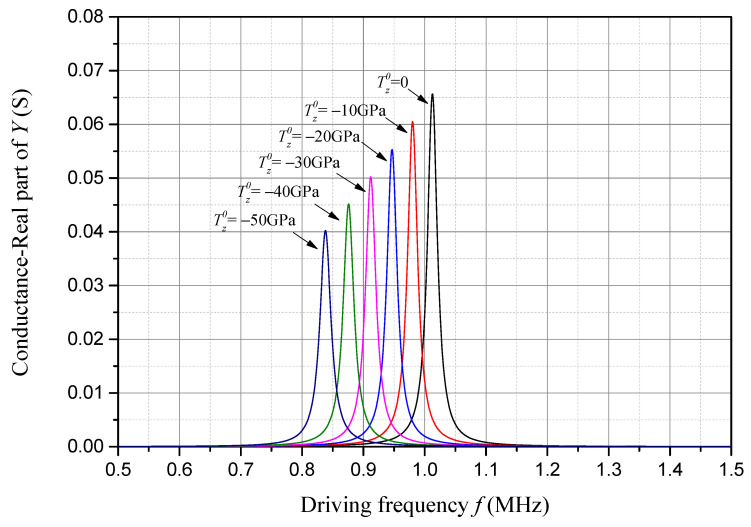
Conductance frequency response considering compressive initial stress.

Conductance is the real part of admittance, and its frequency response considering different compressive initial stresses is presented in Figure 10. The frequency corresponding to the peak value of the conductance is also approximately the resonant frequency of the sample piezoelectric plate. As one can see in Figure 10, with an increase in the compressive initial stress, both the conductance peak and the corresponding resonant frequency decrease linearly.

**Figure 11:**
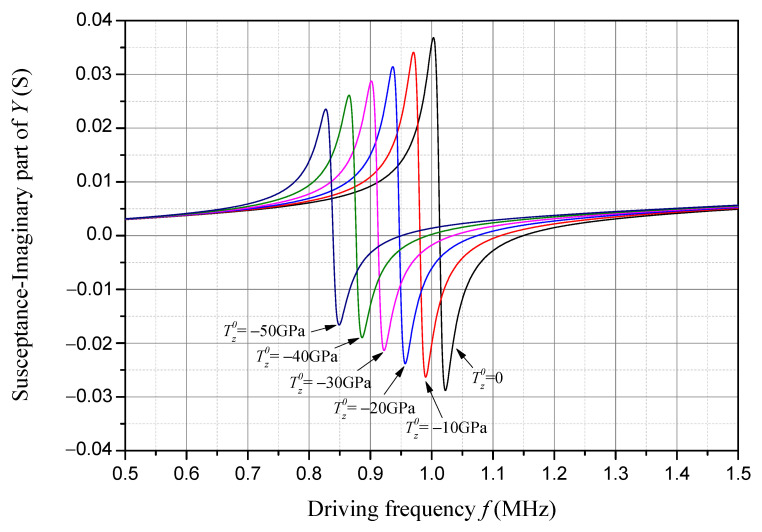
Susceptance frequency response considering compressive initial stress.

Susceptance is the imaginary part of admittance, and its frequency response considering different compressive initial stresses is presented in Figure 11. The frequencies where the susceptance firstly and secondly approaches zero are, respectively, the exact resonant and anti-resonant frequencies of the sample piezoelectric plate. As one can see in Figure 11, with an increase in the compressive initial stress, the resonant frequency, the anti-resonant frequency, and the absolute peak and valley values of the susceptance decrease linearly.

### 4.2. The Sensitivities of Different Characterizations to Compressive Initial Stress and Procedures to Identify Static Stress

The approximate linear relationship between initial stress increasing and impedance characterizations changing are observed in Section 4.1. The change rate of the characterizations with respect to the compressive initial stress is considered to be the sensitivity of the characterizations to the compressive initial stress.

**Figure 12:**
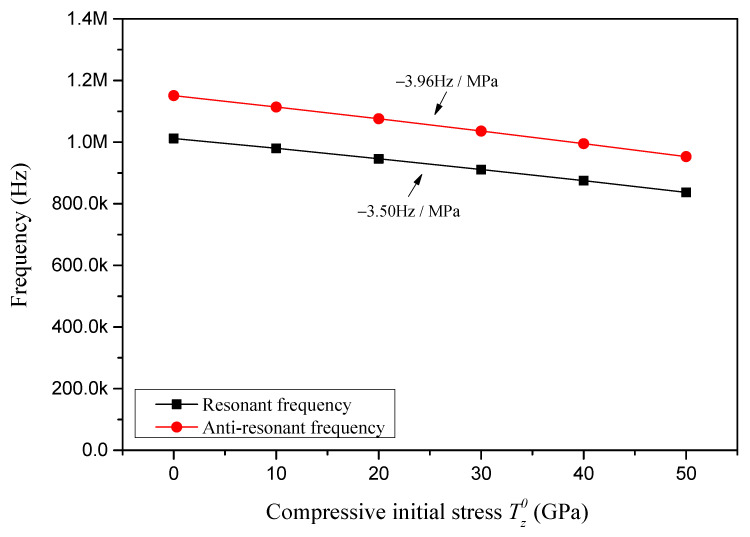
Resonant and anti-resonant frequencies with respect to compressive initial stress.

For the sample piezoelectric plate employed in Section 4.1, the resonant and anti-resonant frequencies with respect to the compressive initial stress are presented in Figure 12. It is observed that with increasing of compressive initial stress, both the resonant and anti-resonant frequencies decrease linearly, and the sensitivities of resonant and anti-resonant frequencies to the compressive initial stress are, respectively, identified as −3.50 Hz/MPa and −3.96 Hz/MPa.

**Figure 13:**
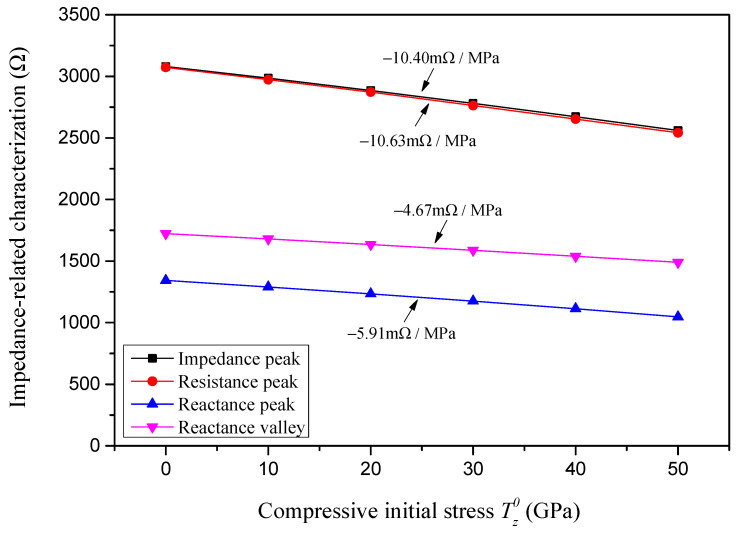
Impedance-related characterizations with respect to compressive initial stress.

The impedance-related characterizations of the sample piezoelectric plate with respect to the compressive initial stress are presented in Figure 13. It is found that with an increase in the compressive initial stress, the peak value of the absolute impedance, the peak value of the resistance, the peak value of the reactance, and the valley value of the reactance all decrease linearly, and the sensitivity of these impedance-related characterizations to the compressive initial stress are, respectively, identified as −10.40 mΩ/MPa, −10.63 mΩ/MPa, −5.91 mΩ/MPa and −4.67 mΩ/MPa.

**Figure 14:**
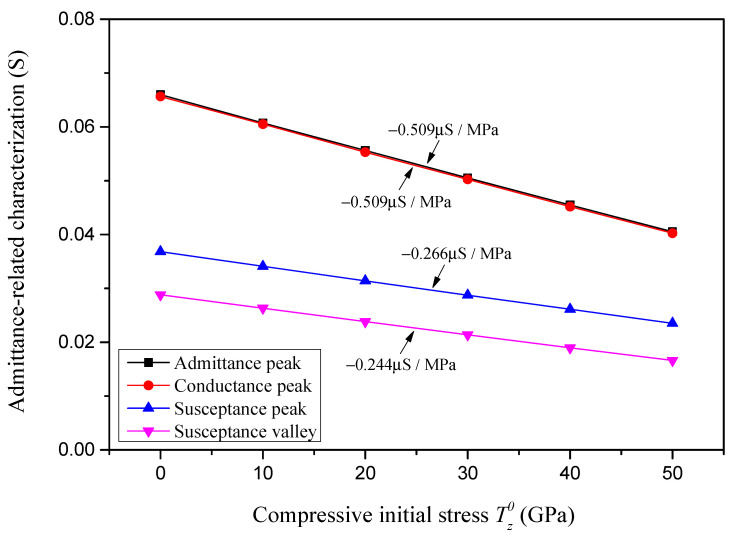
Admittance-related characterizations with respect to compressive initial stress.

The admittance-related characterizations of the sample piezoelectric plate in relation to the compressive initial stress are presented in Figure 14. It is shown that with an increase in the compressive initial stress, the peak value of the absolute admittance, the peak value of the conductance, the peak value of the susceptance, and the valley value of the susceptance all decrease linearly, and the sensitivity of these admittance-related characterizations to the compressive initial stress are, respectively, identified as −0.509 μS/MPa, −0.509 μS/MPa, −0.266 μS/MPa and −0.244 μS/MPa.

Note that the identified sensitivities of the characterizations are the slope of the linear fitting line of the scatters.

From the above sensitivity analysis, we should notice that the impedance-related characterizations that are more sensitive to the changes in initial stress are the peak value of the absolute impedance, the peak value of the resistance, and their corresponding anti-resonant frequency. As for the admittance-related characterizations, the ones that are more sensitive to changes in the initial stress are the peak value of the absolute admittance, the peak value of the conductance, and their corresponding resonant frequency.

To effectively identify the initial stress utilizing the sensitivity of the above-mentioned characterizations to the initial stress, we need to refer to the original characterizations at zero initial stress. Since the original characterizations may suffer noise in practice, the change rate of the characterizations compared to the original characterizations at zero initial stress is also an important indicator reflecting the potential to identify the initial stress.

**Figure 15:**
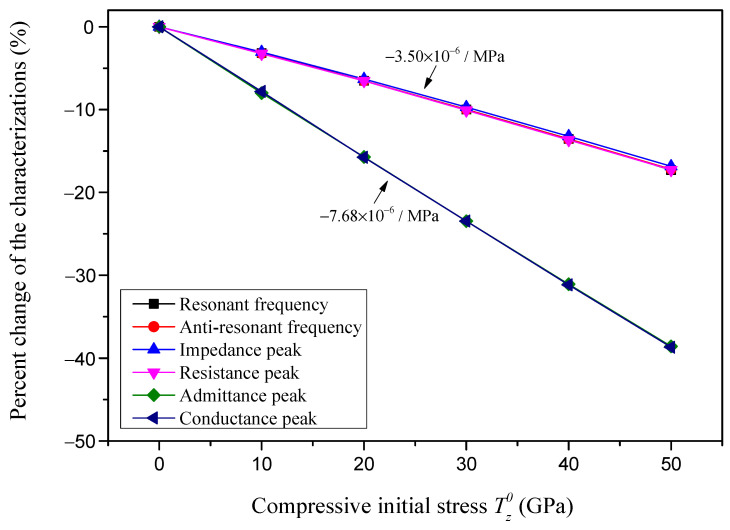
The percent change in the characterizations with respect to the compressive initial stress.

Taking the characterizations at zero initial stress as the baseline, the relative change rate of the characterizations with respect to the compressive initial stress is defined as the relative sensitivity. The percent change in the characterizations for different compressive initial stresses are presented in Figure 15, and the relative sensitivities of different characterizations are also marked in Figure 15. It is found that the relative sensitivity of both the absolute admittance peak and the conductance peak are very close to each other and identified as approximately −7.68 × 10^−6^/MPa. Meanwhile, the relative sensitivity of the other potential characterizations, i.e., the resonant frequency, anti-resonant frequency, the absolute impedance peak and the resistance peak, are approximately identified as −3.50 × 10^−6^/MPa.

Considering both the sensitivity and the relative sensitivity of the potential characterizations to the initial stress, the absolute admittance peak, the conductance peak, and their corresponding resonant frequency should have the most potential in identifying the initial stress. The second potential characterizations should be the absolute impedance peak, the resistance peak, and their corresponding anti-resonant frequency.

Taking the peak value of admittance as an illustrative example, the internal static stress of structures can be identified through the following procedures. First, encapsulate the piezoelectric plate with proper materials dependent on the application environment, and calibrate the original admittance peak value of the encapsulated piezoelectric plate under no load. Next, assess the admittance peak value of the encapsulated piezoelectric plate under a specified static load, comparing it with the original admittance peak value to calibrate the sensitivity of the admittance peak value to variations in the initial stress. Subsequently, embed the encapsulated piezoelectric plate within the target structure and adjust its orientation to align with the target stress. Finally, measure the admittance peak value of the embedded piezoelectric plate; the internal static stress can be obtained by dividing the difference in admittance peak values by its sensitivity to the initial stress.

Reviewing Figure 9, Figure 10, Figure 14 and Figure 15, the absolute admittance peak and the conductance peak are actually very close to each other, and the conductance curve has only one peak, while the admittance curve has both a peak and a valley. Similar to the relation between the absolute admittance peak and the conductance peak, the resistance peak is also very close to the absolute impedance peak. For the sake of simplicity, we shall concentrate on the conductance peak and resistance peak, along with their associated resonant and anti-resonant frequencies, in the following discussion concerning factors that influence sensitivity.

### 4.3. Influence of Geometric Dimensions on Sensitivities of Conductance Peak and Resistance Peak

Through the sensitivity analysis in Section 4.2, the conductance peak and resistance peak are found to be, respectively, the most and second most promising characterizations in identifying the initial stress. In this part, the influence of geometric dimensions on the sensitivities of both the conductance peak and resistance peak in response to compressive initial stress is investigated. The results obtained in this part can help with the design and optimization of PZT-based static stress sensors. In the following discussion, the external constraint stiffness is considered to be *K* = 0, the external viscous damping coefficient is considered to be *C* = 0, and the internal complex damping factor is considered to be *Q* = 0.02.

**Figure 16:**
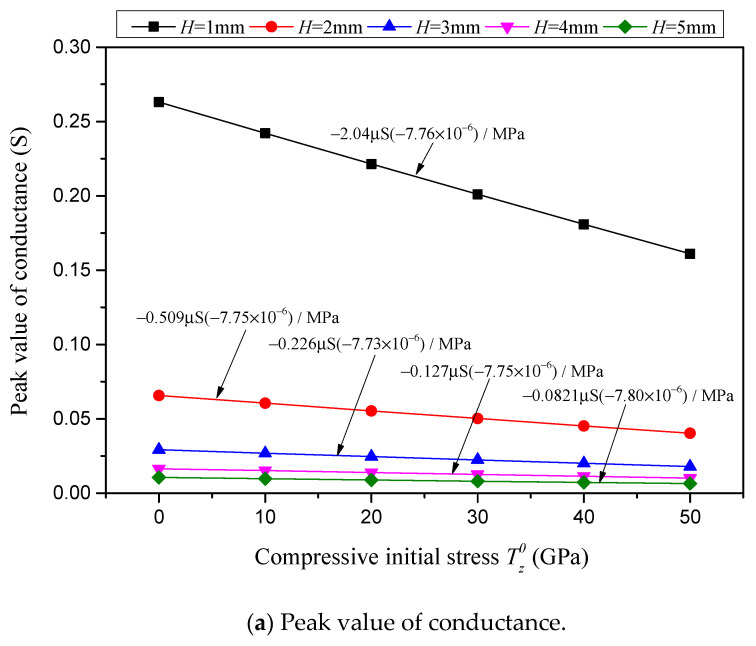

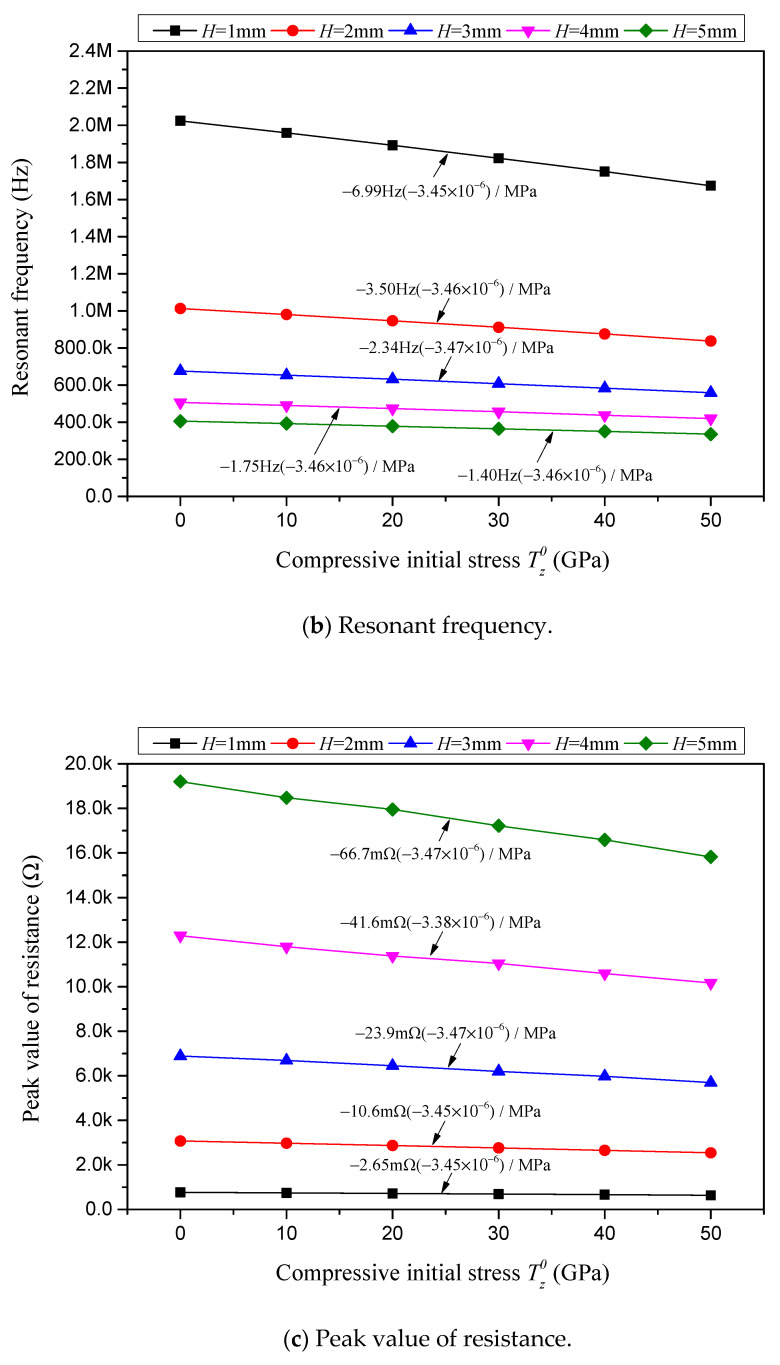

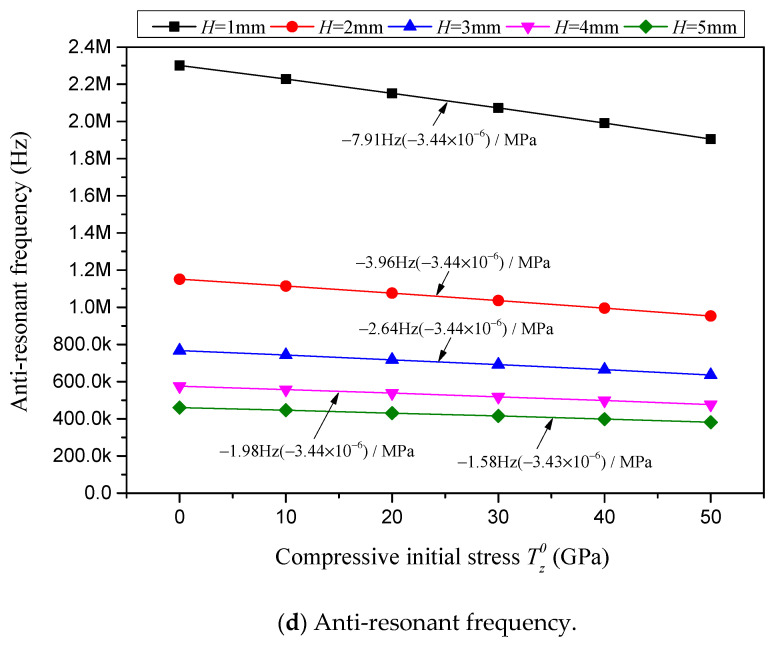
Characterizations with respect to compressive initial stress considering different piezoelectric layer thicknesses *H* while the cross-sectional area is *a =* 100 mm^2^.

The conductance peak value, resonant frequency, resistance peak value and anti-resonant frequency, in relation to the compressive initial stress for various piezoelectric layer thickness *H*, are presented, respectively, in Figure 16a–d. The scatters presented in Figure 16 are obtained based on sample piezoelectric plates with a constant cross-sectional area of *a* = 100 mm^2^ and various thicknesses of *H* = 1 mm, 2 mm, 3 mm, 4 mm and 5 mm. The sensitivities of the conductance peak value, resonant frequency, resistance peak value and anti-resonant frequency to the compressive initial stress for various piezoelectric layer thicknesses are marked in Figure 16 and summarized in Table 3.

**Figure 17:**
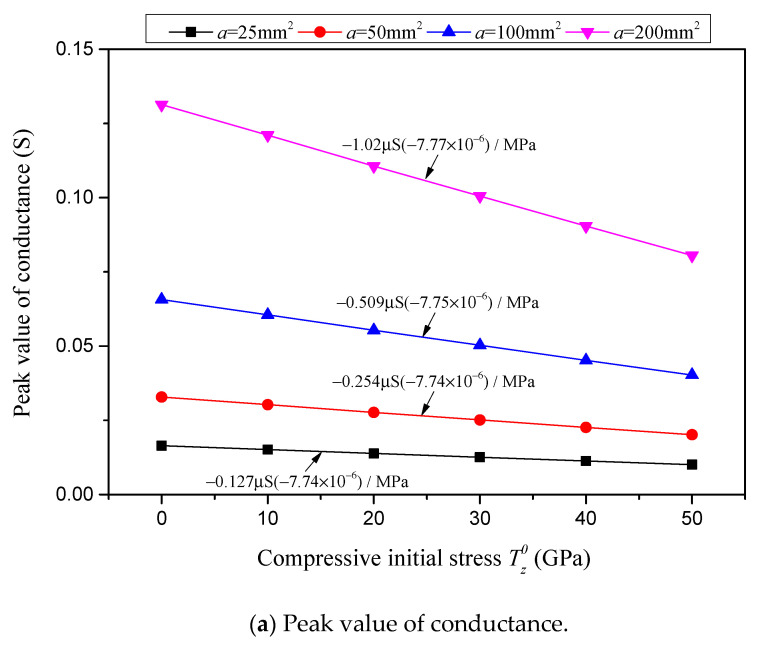

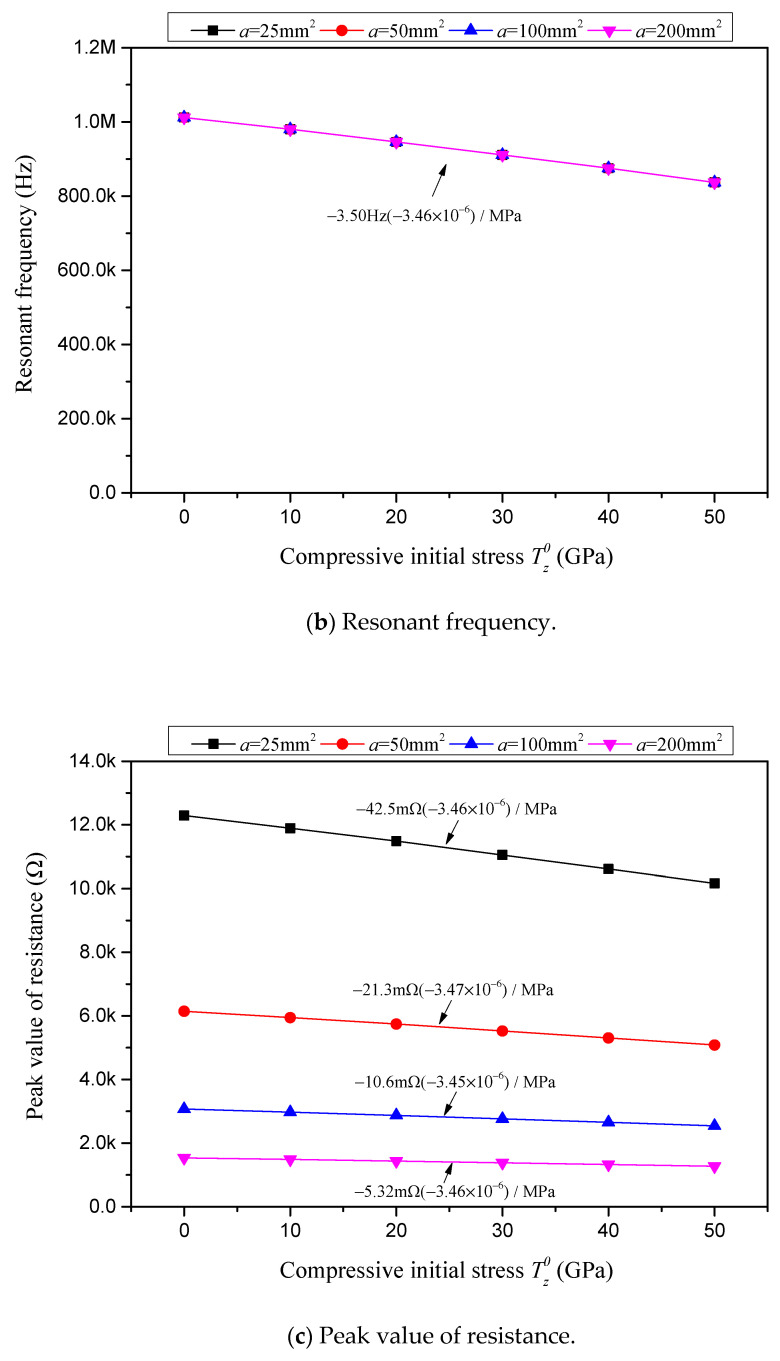

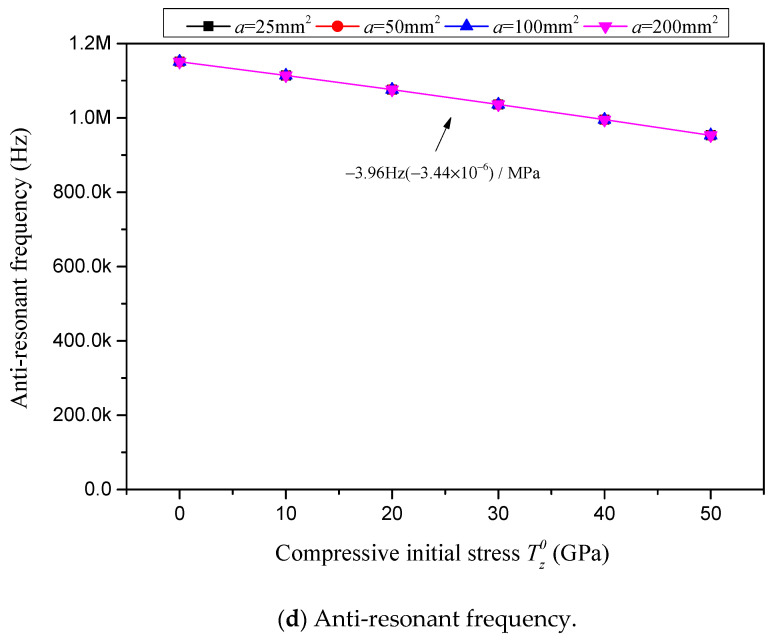
Characterizations with respect to compressive initial stress considering different piezoelectric layer cross-sectional areas *a* while the thickness is *H* = 2 mm.

Similarly, the conductance peak value, resonant frequency, resistance peak value and anti-resonant frequency, in relation to the compressive initial stress for various piezoelectric layer cross-sectional areas *a*, are presented, respectively, in Figure 17a–d. Figure 17 is obtained based on sample piezoelectric plates with a constant thickness of *H* = 2 mm and various cross-sectional areas of *a* = 25 mm^2^, 50 mm^2^, 100 mm^2^ and 200 mm^2^. The sensitivities of the potential characterizations to the compressive initial stress for various piezoelectric layer cross-sectional areas are marked in Figure 17 and summarized in Table 3.

In Figure 16a and Figure 17a and Table 3, it is found that the sensitivity of the conductance peak value in response to the compressive initial stress is significantly affected by the geometric dimensions of the piezoelectric layer. With an increase in the piezoelectric layer thickness *H*, the sensitivity of the conductance peak value diminishes significantly. Notably, when the thickness is quintupled from 1 mm to 5 mm, the sensitivity reduces from −2.04 μS/MPa to a mere 1/25 of its original value, yielding −0.0821 μS/MPa. Conversely, as the cross-sectional area of the piezoelectric layer expands, the sensitivity of the conductance peak value rises in a linear fashion. Specifically, when the cross-sectional area is enlarged eightfold from 25 mm^2^ to 200 mm^2^, the sensitivity correspondingly increases eightfold, shifting from−0.127 μS/MPa to −1.02 μS/MPa. In contrast to the sensitivity term, the relative sensitivity of the conductance peak value remains unaffected by the geometric dimensions and maintains a constant level, at approximately −7.75 × 10^−6^/MPa.

Although the sensitivity of the resistance peak value in response to compressive initial stress is also greatly affected by the geometric dimensions, as one can notice in Figure 16c and Figure 17c and Table 3, the influence patten is completely different. Unlike the sensitivity of the conductance peak value, as the piezoelectric layer thickness increases, the sensitivity of the resistance peak value increases significantly. Specifically, when the thickness is quintupled from 1 mm to 5 mm, the sensitivity increases twenty-five times from −2.65 mΩ/MPa to −66.7 mΩ/MPa. Conversely, as the cross-sectional area of the piezoelectric layer expands, the sensitivity of the resistance peak value reduces linearly. Specifically, when the cross-sectional area is enlarged eightfold from 25 mm^2^ to 200 mm^2^, the sensitivity correspondingly reduces to 1/8 of the original value from−42.5 mΩ/MPa to −5.32 mΩ/MPa. Similarly to the relative sensitivity of the conductance peak value, the relative sensitivity of the resistance peak value also remains unaffected by the geometric dimensions and maintains a constant level, at approximately −3.45 × 10^−6^/MPa.

As for the influence of geometric dimensions on the sensitivities of the resonant and anti-resonant frequencies presented in Figure 16b,d and Figure 17b,d and Table 3, the sensitivities of both the resonant and anti-resonant frequencies are not affected by the change in the piezoelectric layer cross-sectional area, but are affected by the variation in the piezoelectric layer thickness. With an increase in the piezoelectric layer thickness, the sensitivities of both the resonant and anti-resonant frequencies reduce linearly. It is noteworthy that the relative sensitivities of both the resonant and anti-resonant frequencies remain unaffected by the geometric dimensions and maintain at a constant level, at approximately −3.45 × 10^−6^/MPa.

The above discussion indicates that when the conductance peak value of an embedded piezoelectric plate is employed to identify the initial stress, enabling the identification of static stress, opting for thin plates with expansive cross-sectional areas is the preferred course of action. Conversely, when the resistance peak value is employed for stress identification, thicker piezoelectric plates with diminished cross-sectional areas exhibit heightened sensitivity. In instances where the resonant and anti-resonant frequencies serve as indicators of stress, the use of thin piezoelectric plates results in commendable sensitivity, with the cross-sectional area having no discernible impact on this sensitivity.

### 4.4. The Influence of the Constraint Stiffness K, the External Viscous Damping Coefficient C and the Internal Complex Damping Factor Q on the Sensitivity of the Conductance Peak and Resistance Peak

In the modeling of an embedded piezoelectric plate, the surrounding environment is simplified to a pair of viscoelastic constraints with a stiffness of *K* and a viscous damping coefficient of *C*. To consider the internal damping effect of the piezoelectric plate, a complex damping factor *Q* is introduced, and the elastic constant $c_{33}^{E}$ is replaced with $c_{33}^{E}\left(1+Qj\right)$. In this part, the influence of *K*, *C* and *Q* on the sensitivities of both the conductance peak and the resistance peak in response to the compressive initial stress is investigated. The findings garnered from this part can help in assessing the efficacy of initial stress identification across diverse application environments. In the following discussion, a sample piezoelectric plate with a cross-sectional aera of $a=100\;{mm}^{2}$ and a thickness of H=2 mm is employed.

**Figure 18:**
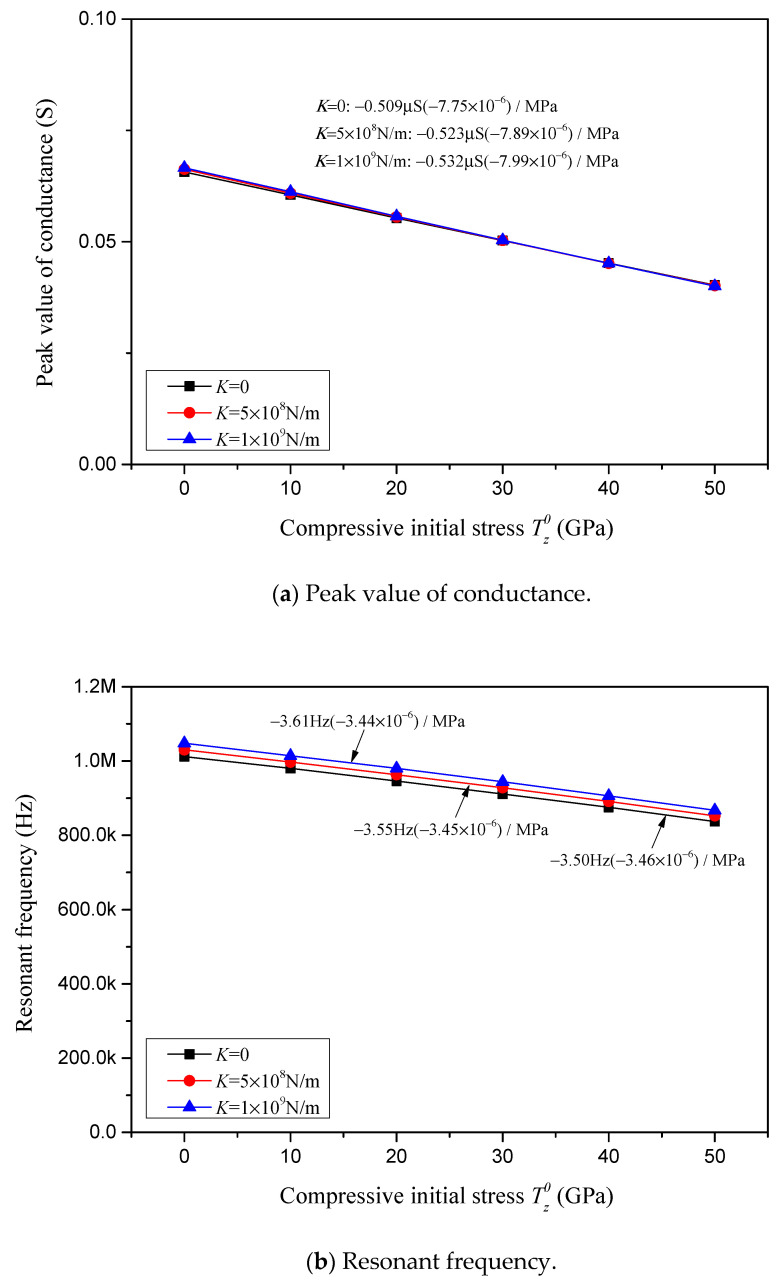

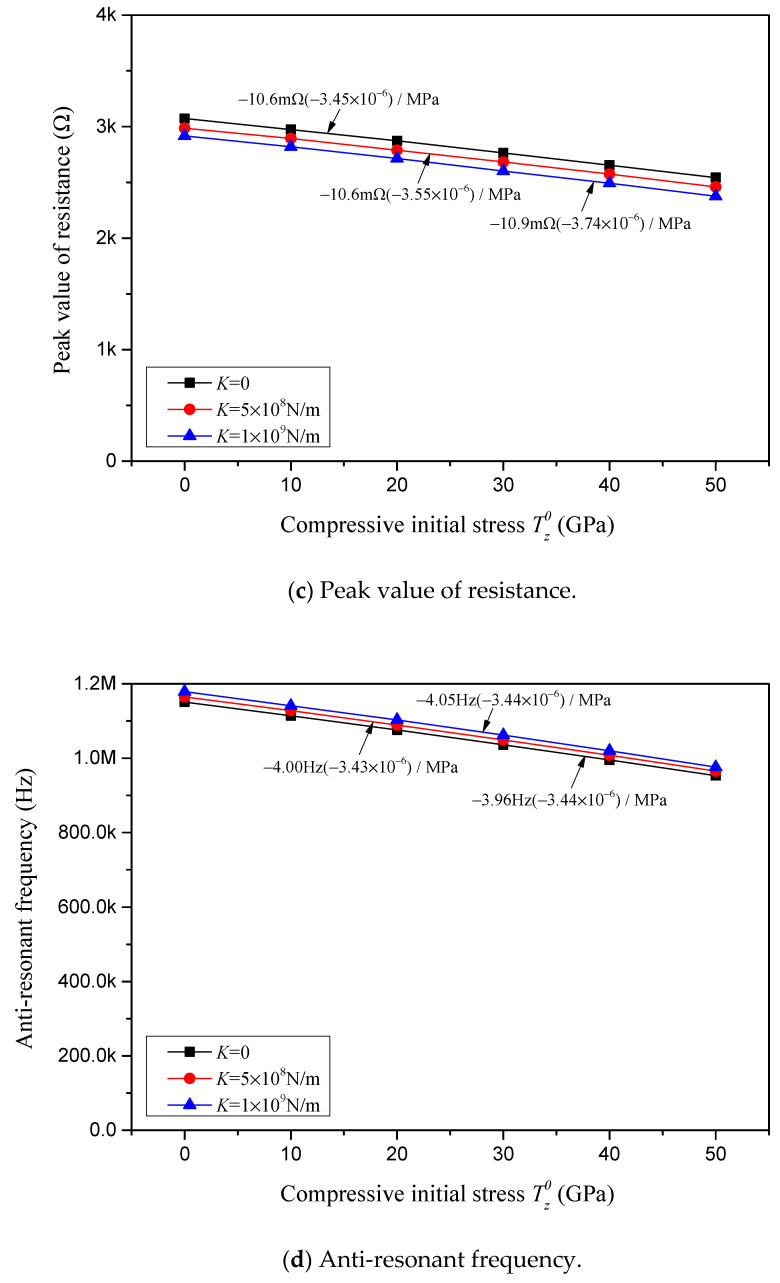
Characterizations with respect to compressive initial stress considering different elastic constraints *K*.

The conductance peak value, resonant frequency, resistance peak value and anti-resonant frequency, in correlation with compressive initial stress for varying constraint stiffnesses *K*, are depicted, respectively, in Figure 18a–d. In these Figures, the viscous damping coefficient is considered to be *C* = 0, the complex damping factor is considered to be *Q* = 0.02 and the various constraint stiffnesses are considered to be *K* = 0, 5 × 10^8^ N/m and 1 × 10^9^ N/m. The sensitivities of the conductance peak value, resonant frequency, resistance peak value and anti-resonant frequency in response to the compressive initial stress for various constraint stiffnesses are marked in Figure 18 and summarized in Table 4.

**Figure 19:**
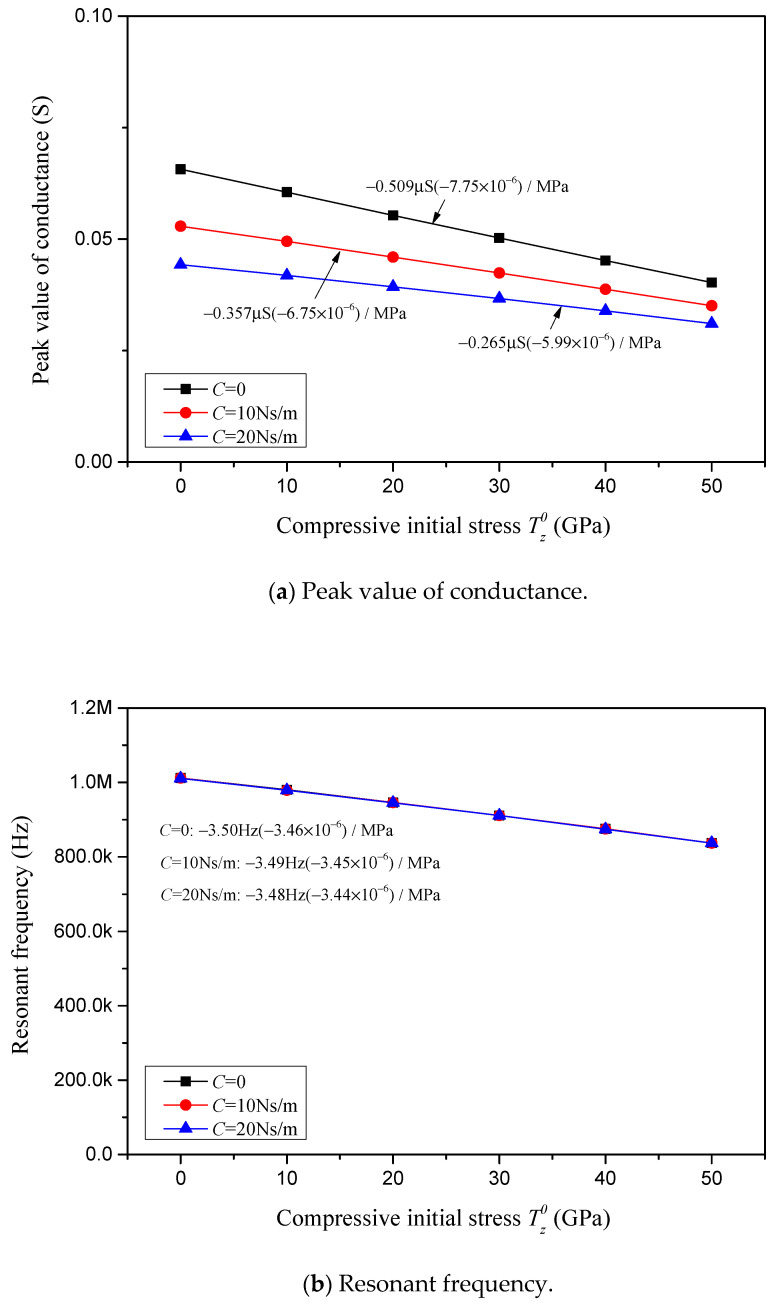

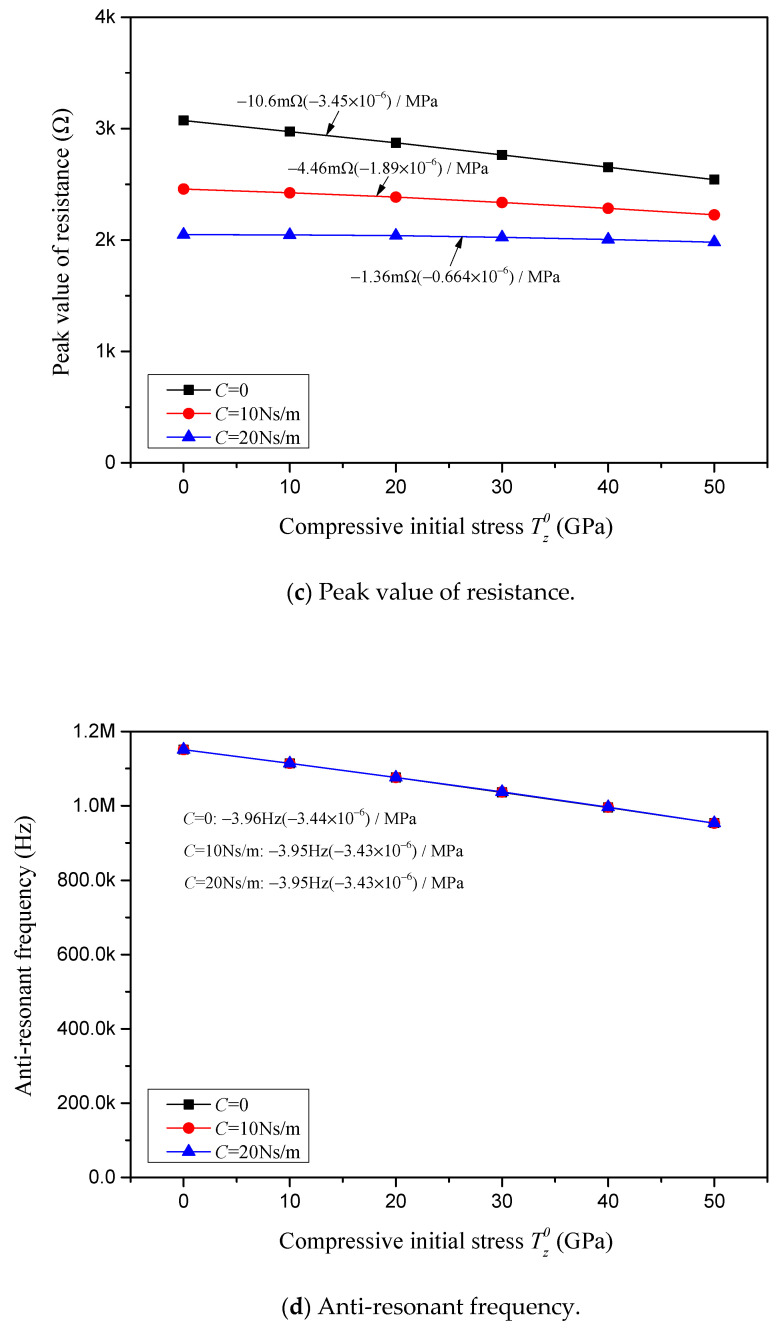
Characterizations with respect to compressive initial stress considering different external viscous damping coefficients *C*.

Similarly, the conductance peak value, resonant frequency, resistance peak value and anti-resonant frequency in correlation with the compressive initial stress for various viscous damping coefficients *C* are depicted, respectively, in Figure 19a–d. In Figure 19, the constraint stiffness is considered to be *K* = 0, the complex damping factor is considered to be *Q* = 0.02 and the varying viscous damping coefficients are considered to be *C* = 0, 10 Ns/m and 20 Ns/m. The sensitivities of the conductance peak value, resonant frequency, resistance peak value and anti-resonant frequency in response to the compressive initial stress for various viscous damping coefficients are marked in Figure 19 and summarized in Table 4.

**Figure 20:**
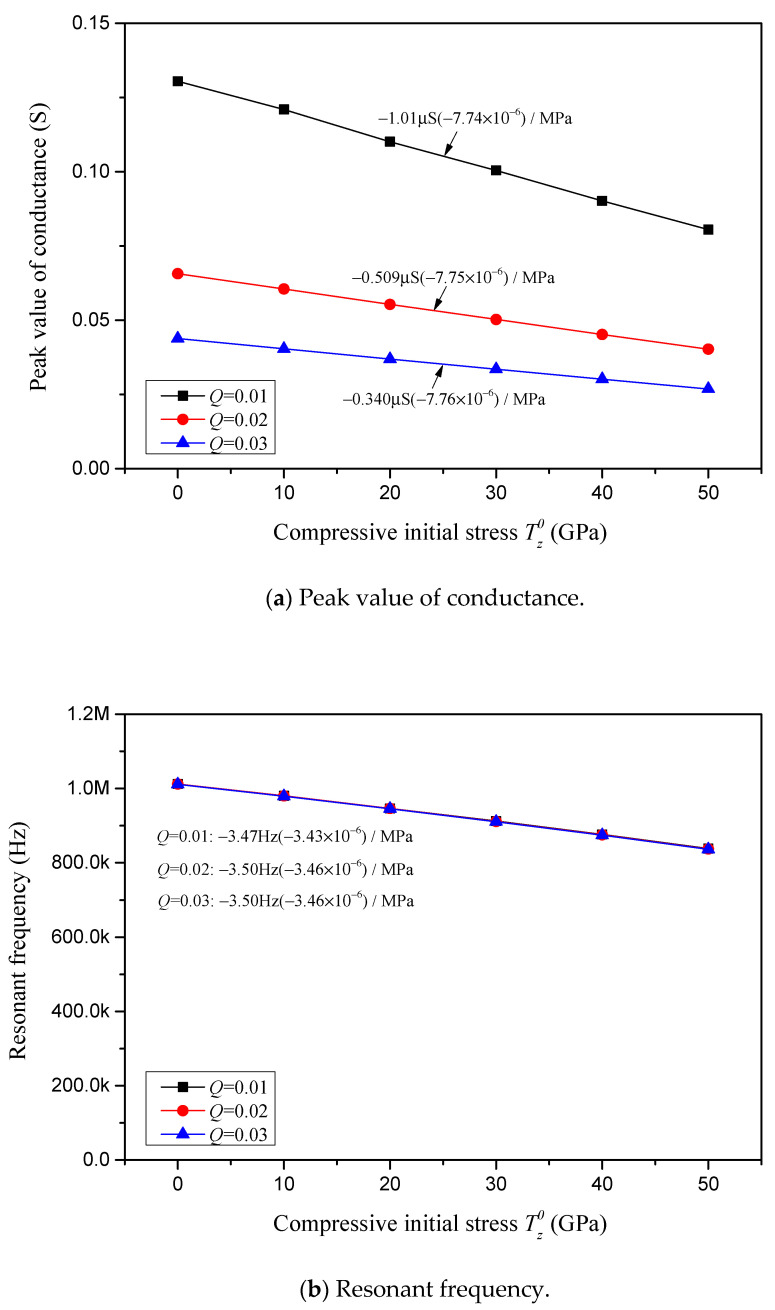

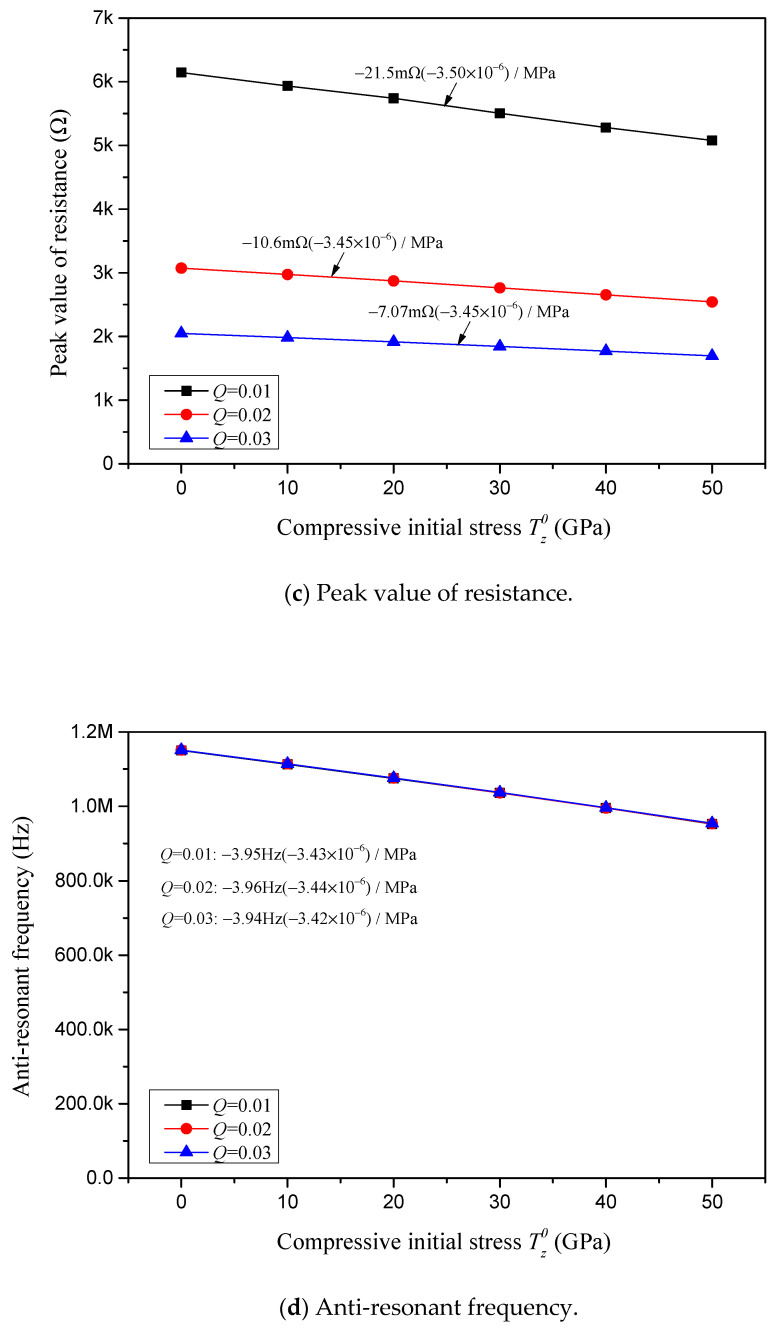
Characterizations with respect to compressive initial stress considering different internal complex damping factors *Q*.

For varying complex damping factors *Q*, the conductance peak value, resonant frequency, resistance peak value and anti-resonant frequency in correlation with the compressive initial stress are depicted, respectively, in Figure 20a–d. In Figure 20, the constraint stiffness is considered to be *K* = 0, the external viscous damping coefficient is considered to be *C* = 0, and the varying internal complex damping factors are considered to be *Q* = 0.01, 0.02 and 0.03. The sensitivities of the conductance peak value, resonant frequency, resistance peak value and anti-resonant frequency in response to the compressive initial stress for varying complex damping factors are marked in Figure 20 and summarized in Table 4.

In Figure 18a, Figure 19a and Figure 20a and Table 4, it is found that the sensitivity of the conductance peak value in response to the compressive initial stress is barely affected by the change in constraint stiffness, but reduces linearly with an increase in the external viscous damping coefficient *C* and internal complex damping factor *Q*. It is noteworthy that the relative sensitivity is not relevant to *K* and *Q*, but is affected by the external damping coefficient *C*, i.e., the relative sensitivity reduces as the external damping coefficient increases.

As one can notice in Figure 18c, Figure 19c and Figure 20c and Table 4, the influence pattern of *K*, *C* and *Q* on the sensitivity of the resistance peak value is similar to that on the sensitivity of the conductance peak value. The resistance peak value in response to the compressive initial stress is barely affected by the change in constraint stiffness, but reduces significantly with an increase in the external viscous damping coefficient *C*, and reduces linearly with an increase in the internal complex damping factor *Q*. It is noteworthy that the relative sensitivity of the resistance peak value is not relevant to *K* and *Q*, but is significantly affected by the external damping coefficient *C*, i.e., the relative sensitivity greatly reduces as the external damping coefficient *C* increases.

Regarding the impact of *K*, *C* and *Q* on the sensitivities of the resonant and anti-resonant frequencies showcased in Figure 18b,d, Figure 19b,d and Figure 20b,d and Table 4, it is observed that both the sensitivities and relative sensitivities of the resonant and anti-resonant frequencies remain invariant with respect to the constraint stiffness *K*, external viscous damping coefficient *C* and internal complex damping factor *Q*. The sensitivities of the resonant and anti-resonant frequencies maintain a constant level, at approximately −3.50 Hz/MPa and −3.95 Hz/MPa, respectively. And the relative sensitivities of the resonant and anti-resonant frequencies maintain the same constant level, at approximately −3.45 × 10^−6^/MPa.

The preceding discussion suggests that when utilizing the conductance peak value or resistance peak value of an embedded piezoelectric plate to identify the initial stress, opting for piezoelectric materials with extremely low damping effects is the most advisable approach. Moreover, the presence of an encapsulating material with a pronounced damping effect may lead to a significant reduction in identification sensitivity. In contrast, when resonant and anti-resonant frequencies are employed as stress indicators, the damping effect of encapsulation becomes negligible.

## 5. Conclusions

In this study, based on the nonlinear elastic dynamic governing equations of piezoelectric materials, using the displacement method, an impedance analysis model of an embedded piezoelectric plate considering the effect of initial stress is formulated and validated through a fundamental experiment and a finite element analysis. An explicit analytical relationship between initial stress and impedance characterizations is offered, and an approximate linear correlation between the increment in initial stress and the change in impedance characterizations is observed in the numerical investigation. The proposed model enabled the performance prediction and design optimization of piezoelectric-based static stress sensors.

The results of the sensitivity and relative sensitivity analyses for various impedance characterizations in response to the initial stress reveal that the absolute admittance peak, the conductance peak and their respective resonant frequencies are highly promising for the identification of initial stress. Following this closely, the absolute impedance peak, the resistance peak and their corresponding anti-resonant frequencies are identified as the second most promising indicators.

Through an examination of the impact of geometric dimensions on sensitivity, it is found that for the purpose of stress identification utilizing the conductance peak value of an embedded piezoelectric plate, the preference lies with thin plates featuring extensive cross-sectional areas. Conversely, for stress sensing using the resistance peak value, the sensitivity is enhanced by employing thicker piezoelectric plates with reduced cross-sectional areas. When resonant and anti-resonant frequencies are used to identify static stress, the sensitivity achieved with thin piezoelectric plates is commendable, and the cross-sectional area has no discernible impact on this sensitivity.

An analysis of the influence of constraint stiffness and damping mechanisms on sensitivity indicates that for stress identification through the conductance or resistance peak values of an embedded piezoelectric plate, the use of piezoelectric materials with an extremely low damping factor is the optimal strategy. Furthermore, the incorporation of an encapsulating material with high damping properties can significantly diminish the identification sensitivity. In contrast, when resonant and anti-resonant frequencies are used as stress indicators, the damping effect of encapsulation becomes negligible.

## Figures and Tables

**Table 1:** Material parameters of the sample PZT disks.

	$f_{r}$ (MHz)	$f_{a}$ (MHz)	$c_{33}^{E}$ (GPa)	$e_{33}$ (C/m^2^)
Sample 1	3.60	4.31	92.1	22.32
Sample 2	3.57	4.28	90.6	22.23
Sample 3	3.70	4.48	97.0	23.75

**Table 2:** One-dimensional material parameters of standard PZT-5H [8,34].

**Density** ρ	$\mathrm{Elastic}\;\mathrm{Constant}\;c_{33}^{E}$	$\mathrm{Piezoelectric}\;\mathrm{Constant}\;e_{33}$	$\mathrm{Dielectric}\;\mathrm{Constant}\;\varepsilon_{33}^{S}$
7500 kg/m^3^	117 GPa	23.3 C/m^2^	1470 × 8.854 pF/m

**Table 3:** Influence of geometric dimensions on sensitivities of conductance peak and resistance peak.

	Conductance Peak Value		Resonant Frequency		Resistance Peak Value		Anti-Resonant Frequency	
	Sensitivity (μS/MPa)	Relative Sensitivity (1/MPa)	Sensitivity (Hz/MPa)	Relative Sensitivity (1/MPa)	Sensitivity (mΩ/MPa)	Relative Sensitivity (1/MPa)	Sensitivity (Hz/MPa)	Relative Sensitivity (1/MPa)
*H* = 1 mm	−2.04	−7.76 × 10^−6^	−6.99	−3.45 × 10^−6^	−2.65	−3.45 × 10^−6^	−7.91	−3.44 × 10^−6^
*H* = 2 mm	−0.509	−7.75 × 10^−6^	−3.50	−3.46 × 10^−6^	−10.6	−3.45 × 10^−6^	−3.96	−3.44 × 10^−6^
*H* = 3 mm	−0.226	−7.73 × 10^−6^	−2.34	−3.47 × 10^−6^	−23.9	−3.47 × 10^−6^	−2.64	−3.44 × 10^−6^
*H* = 4 mm	−0.127	−7.75 × 10^−6^	−1.75	−3.46 × 10^−6^	−41.6	−3.38 × 10^−6^	−1.98	−3.44 × 10^−6^
*H* = 5 mm	−0.0821	−7.80 × 10^−6^	−1.40	−3.46 × 10^−6^	−66.7	−3.47 × 10^−6^	−1.58	−3.43 × 10^−6^
*a* = 25 mm^2^	−0.127	−7.74 × 10^−6^	−3.50	−3.46 × 10^−6^	−42.5	−3.46 × 10^−6^	−3.96	−3.44 × 10^−6^
*a* = 50 mm^2^	−0.254	−7.74 × 10^−6^	−3.50	−3.46 × 10^−6^	−21.3	−3.47 × 10^−6^	−3.96	−3.44 × 10^−6^
*a* = 100 mm^2^	−0.509	−7.75 × 10^−6^	−3.50	−3.46 × 10^−6^	−10.6	−3.45 × 10^−6^	−3.96	−3.44 × 10^−6^
*a* = 200 mm^2^	−1.02	−7.77 × 10^−6^	−3.50	−3.46 × 10^−6^	−5.32	−3.46 × 10^−6^	−3.96	−3.44 × 10^−6^

**Table 4:** Influence of the elastic constraint *K*, external viscous damping coefficient *C* and internal complex damping factor *Q* on sensitivities of conductance peak and resistance peak.

	Conductance Peak Value		Resonant Frequency		Resistance Peak Value		Anti-Resonant Frequency	
	Sensitivity (μS/MPa)	Relative Sensitivity (1/MPa)	Sensitivity (Hz/MPa)	Relative Sensitivity (1/MPa)	Sensitivity (mΩ/MPa)	Relative Sensitivity (1/MPa)	Sensitivity (Hz/MPa)	Relative Sensitivity (1/MPa)
*K* = 0	−0.509	−7.75 × 10^−6^	−3.50	−3.46 × 10^−6^	−10.6	−3.45 × 10^−6^	−3.96	−3.44 × 10^−6^
*K* = 5 × 10^8^ N/m	−0.523	−7.89 × 10^−6^	−3.55	−3.45 × 10^−6^	−10.6	−3.55 × 10^−6^	−4.00	−3.43 × 10^−6^
*K* = 1 × 10^9^ N/m	−0.532	−7.99 × 10^−6^	−3.61	−3.44 × 10^−6^	−10.9	−3.74 × 10^−6^	−4.05	−3.44 × 10^−6^
C = 0	−0.509	−7.75 × 10^−6^	−3.50	−3.46 × 10^−6^	−10.6	−3.45 × 10^−6^	−3.96	−3.44 × 10^−6^
*C* = 10 Ns/m	−0.357	−6.75 × 10^−6^	−3.49	−3.45 × 10^−6^	−4.46	−1.89 × 10^−6^	−3.95	−3.43 × 10^−6^
*C* = 20 Ns/m	−0.265	−5.99 × 10^−6^	−3.48	−3.44 × 10^−6^	−1.36	−0.66 × 10^−6^	−3.95	−3.43 × 10^−6^
*Q* = 0.01	−1.01	−7.74 × 10^−6^	−3.47	−3.43 × 10^−6^	−21.5	−3.50 × 10^−6^	−3.95	−3.43 × 10^−6^
*Q* = 0.02	−0.509	−7.75 × 10^−6^	−3.50	−3.46 × 10^−6^	−10.6	−3.45 × 10^−6^	−3.96	−3.44 × 10^−6^
*Q* = 0.03	−0.340	−7.76 × 10^−6^	−3.50	−3.46 × 10^−6^	−7.07	−3.45 × 10^−6^	−3.94	−3.42 × 10^−6^

## Data Availability

The datasets used for generating the plots and results in the present study can be directly obtained from equations provided in the main text and the COMSOL project provided in the Supplementary Materials.

## Supplementary Materials

The following supporting information can be downloaded at: https://www.mdpi.com/article/10.3390/s24217096/s1, COMSOL project employed in Section 3.2 for model validation.

## Notation List

a	Effective cross-sectional area
C	External viscous damping coefficient
$c_{33}^{D}$	Elastic stiffnesses at constant electric displacement
$c_{33}^{E}$	Elastic stiffness at constant electric field (superscript *E* is dropped in Section 2)
$D_{z}$	Electric displacement component along *z* direction
$E_{z}$	Electric field strength component along *z* direction
e	Euler number
$e_{33}$	Piezoelectric constant for e-type constitutive equations
f	Input voltage frequency (driving frequency)
$f_{a}$	Anti-resonant frequency
$f_{r}$	Resonant frequency
H	Thickness of piezoelectric plate
h	Semi-thickness of piezoelectric plate
$h_{33}$	Piezoelectric constant for h-type constitutive equations
$I_{in}$	Input current amplitude
i	Input current
j	Imaginary unit
K	External constraint stiffness
$k_{t}$	Thickness mode coupling factor
Q	Internal complex damping factor
q	Input charge
$S_{z}$	Strain component along *z* direction
$T_{z}$	Stress component along *z* direction
$T_{z}^{0}$	Initial stress along z direction
t	Time
$V_{in}$	Input voltage amplitude
v	Input voltage
$v^{D}$	Longitudinal wave velocity
*w*	Displacement along *z* direction
α	$\sqrt{\rho \omega^{2}/\left(\eta +T_{z}^{0}\right)}$
β	$e_{33}/\varepsilon_{33}^{S}$
δ	${e_{33}}^{2}/\varepsilon_{33}^{S}$
$\varepsilon_{33}^{S}$	Dielectric constant at constant strain (superscript *S* is dropped in Section 2)
η	$\left({e_{33}}^{2}+c_{33}^{E}\varepsilon_{33}^{S}\right)/\varepsilon_{33}^{S}$
ρ	Mass density
*ϕ*	Electric potential
ω	Circular frequency of input voltage

## Appendix A

The h-type constitutive equations of a piezoelectric plate polarized along the *z* axis can be written as

(A1) $$\left\{\begin{array}{l} T_{z}=c_{33}^{D}S_{z}-h_{33}D_{z}\\ E_{z}=-h_{33}S_{z}+\varepsilon_{33}^{S}D_{z} \end{array}\right.$$

where $c_{33}^{D}$ denotes the elastic stiffnesses at constant electric displacement, and $h_{33}$ denotes piezoelectric constant for h-type constitutive equations. Based on h-type constitutive equations, the velocity of a longitudinal wave propagating along *z* and the thickness mode coupling factor are obtained as follows:

(A2) $$\left\{\begin{array}{l} v^{D}=\sqrt{\frac{c_{33}^{D}}{\rho }}\\ k_{t}=h_{33}\sqrt{\frac{\varepsilon_{33}^{S}}{c_{33}^{D}}} \end{array}\right.$$

The longitudinal wave velocity $v^{D}$ and the thickness mode coupling factor $k_{t}$ are also related to the resonant and anti-resonant frequencies of a piezoelectric plate as follows:

(A3) $$\left\{\begin{array}{l} f_{a}=\frac{v^{D}}{2t}\\ {k_{t}}^{2}=\frac{\pi }{2}\frac{f_{r}}{f_{a}}\cot\left(\frac{\pi }{2}\frac{f_{r}}{f_{a}}\right) \end{array}\right.$$

Obviously, once $f_{r}$ and $f_{a}$ are known, $k_{t}$ and $v^{D}$ can be easily obtained using Equation (A3). Subsequently, $c_{33}^{D}$ and $h_{33}$ can be solved using Equation (A2). If we compare the h-type constitutive equations with e-type constitutive equations, $c_{33}^{E}$ and $e_{33}$ can be solved as follows:

(A4) $$\left\{\begin{array}{l} c_{33}^{E}=c_{33}^{D}-{h_{33}}^{2}\varepsilon_{33}^{S}\\ e_{33}=h_{33}\varepsilon_{33}^{S} \end{array}\right.$$

All the equations used in Appendix A can be found in reference [33].
